# Glutathione Is Required for the Early Alert Response and Subsequent Acclimation in Cadmium-Exposed *Arabidopsis thaliana* Plants

**DOI:** 10.3390/antiox11010006

**Published:** 2021-12-21

**Authors:** Jana Deckers, Sophie Hendrix, Els Prinsen, Jaco Vangronsveld, Ann Cuypers

**Affiliations:** 1 Centre for Environmental Sciences, Hasselt University, Agoralaan Building D, 3590 Diepenbeek, Belgium; jana.deckers@uhasselt.be (J.D.); sophie.hendrix@uhasselt.be (S.H.); jaco.vangronsveld@uhasselt.be (J.V.); 2 Institute of Crop Science and Resource Conservation (INRES), University of Bonn, 53113 Bonn, Germany; 3 Department of Biology, University of Antwerp, Groenenborgerlaan 171, 2020 Antwerpen, Belgium; els.prinsen@uantwerpen.be

**Keywords:** *Arabidopsis thaliana*, 1-aminocyclopropane-1-carboxylic acid, cadmium, *cadmium-sensitive* *2-1* mutant, ethylene, glutathione, hydrogen peroxide, oxidative challenge

## Abstract

Pollution by cadmium (Cd) is a worldwide problem, posing risks to human health and impacting crop yield and quality. Cadmium-induced phytotoxicity arises from an imbalance between antioxidants and pro-oxidants in favour of the latter. The Cd-induced depletion of the major antioxidant glutathione (GSH) strongly contributes to this imbalance. Rather than being merely an adverse effect of Cd exposure, the rapid depletion of root GSH levels was proposed to serve as an alert response. This alarm phase is crucial for an optimal stress response, which defines acclimation later on. To obtain a better understanding on the importance of GSH in the course of these responses and how these are defined by the rapid GSH depletion, analyses were performed in the GSH-deficient *cadmium-sensitive 2-1* (*cad2-1*) mutant. Cadmium-induced root and leaf responses related to oxidative challenge, hydrogen peroxide (H_2_O_2_), GSH, ethylene, and 1-aminocyclopropane-1-carboxylic acid (ACC) were compared between wild-type (WT) and mutant *Arabidopsis thaliana* plants. Although the *cad2-1* mutant has significantly lower GSH levels, root GSH depletion still occurred, suggesting that the chelating capacity of GSH is prioritised over its antioxidative function. We demonstrated that responses related to GSH metabolism and ACC production were accelerated in mutant roots and that stress persisted due to suboptimal acclimation. In general, the redox imbalance in *cad2-1* mutant plants and the lack of proper transient ethylene signalling contributed to this suboptimal acclimation, resulting in a more pronounced Cd effect.

## 1. Introduction

Cadmium (Cd) is a widespread pollutant. Besides being highly toxic for humans, it is also known to be phytotoxic [1,2]. Even though Cd is a non-essential element for plants, its similarity to essential trace elements like zinc (Zn), calcium (Ca), iron (Fe), and manganese (Mn) enables it to, opportunistically, move along transporters of these essential nutrients [3]. Therefore, plants readily take up Cd and form a gateway to introduce and allow bio-accumulation of Cd in the human food chain, hence posing a risk to human health [4]. Once taken up by the plant, Cd causes the indirect increase of reactive oxygen species (ROS) by tipping the redox balance in favour of pro-oxidants, resulting in an oxidative challenge. One driving force of this imbalance is the depletion of the major antioxidative metabolite glutathione (GSH) [5,6]. This ubiquitous tripeptide is synthesised in two ATP-dependent steps, starting with the formation of γ-glutamylcysteine from cysteine and γ-glutamate, which is catalysed by γ-glutamate-cysteine ligase (GSH1). Subsequently, glycine is attached by glutathione synthetase (GSH2) to form GSH [7]. The antioxidative capacity of GSH arises from its ability to reduce hydrogen peroxide (H_2_O_2_) directly, but mainly through the ascorbate (AsA)-GSH cycle, wherein GSH functions to recycle the antioxidant AsA. Besides its role as major antioxidant, GSH is important in the chelation of Cd, as Cd displays a high affinity towards the sulfhydryl group on the cysteine residue in GSH [8]. Consequently, GSH can directly chelate Cd via its thiol group, but also serves as building block for phytochelatins (PCs), which are oligomers that consist of 2 to 11 γ-Glu-Cys moieties derived from GSH. These oligomers are able to chelate multiple Cd ions and, subsequently, translocate and sequester them in the vacuole, preventing further harm to cysteine-rich functional and structural proteins and Cd-induced ROS generation [8,9]. It was previously demonstrated that during the early responses to Cd stress, PC synthesis is stimulated in roots of *Arabidopsis thaliana* (L.) Heynh. plants. Inevitably, the rapid allocation of GSH to the formation of PCs causes a depletion in root GSH levels, impairing its antioxidative capacity [5,10]. This Cd-induced depletion was shown to be counteracted by increased transcription of *GSH1* [10]. The GSH-deficient, *cadmium-sensitive 2-1* (*cad2-1*) mutant holds a mutation in this *GSH1* gene, resulting in a deletion close to the active site of the encoded enzyme, which catalyses the rate-limiting step in GSH biosynthesis. As a result, this mutant harbours only 30–45% of wild-type (WT) GSH levels in its cells and displays a higher sensitivity towards Cd [11,12].

Another key player in the Cd-induced stress responses is ethylene. This phytohormone is involved in multiple physiological and developmental processes and is considered a central mediator of responses to environmental stressors [13]. The synthesis pathway of ethylene encompasses three sequential steps. Firstly, methionine is converted to S-adenosyl-methionine (SAM) in an ATP-consuming reaction catalysed by SAM synthetase. During the second, rate-limiting step, methylthioadenine (MTA) is removed by 1-aminocyclopropane-1-carboxylic acid (ACC) synthase (ACS), resulting in the production of ACC. Finally, the oxidation of ACC, catalysed by ACC oxidase (ACO), results in the formation of ethylene along with CO_2_ and cyanide (HCN) [14]. While the major regulatory mechanisms of ethylene biosynthesis are situated at the level of ACC production by ACS, the conversion of ACC to ethylene is also controlled by the formation of conjugated ACC (ACCc), which regulates the pool of available ACC [15,16]. In *A. thaliana*, Cd-induced increases in ACC and ethylene synthesis were shown to be mainly attributed to isoforms ACS2 and ACS6, as Cd exposure did not enhance ethylene production in the *acs2-1acs6-1* double knockout mutant [17]. Due to the presence of mitogen-activated protein kinase (MAPK) target sites in their C-terminal domain, both isoforms can be phosphorylated by MPK3 and MPK6, increasing their half-life [14]. In addition, these MAPKs can activate WRKY33, which functions as a transcriptional activator of *ACS2* and *ACS6*. Furthermore, it was previously shown that oxidative signal-inducible kinase 1 (OXI1) becomes induced by a wide range of H_2_O_2_-generating stimuli, including Cd [18]. This kinase is required for full activation of MPK3 and MPK6 and links the oxidative burst to downstream signalling responses [19]. In Cd-exposed *A. thaliana* plants, Cd causes the activation of ROS-generating NADPH oxidases, which further contributes to a redox imbalance in favour of pro-oxidants. These NADPH oxidases are also named respiratory burst oxidase homologues (RBOHs), and *RBOHC*, *RBOHD*, and *RBOHF* are known to be Cd-responsive [20]. Schellingen et al. (2015) demonstrated that ethylene signalling mediates the oxidative challenge by means of, amongst others, activation of *RBOHC* [21]. In the same study, it was demonstrated that ethylene signalling is required for the stimulation of leaf GSH levels under Cd exposure. Multiple other studies support the crosstalk between ethylene and GSH. For example, Yoshida et al. (2009) suggested that ethylene increased GSH biosynthesis in ozone-exposed *A. thaliana* plants, while Chen et al. (2013) demonstrated that ROS production induced by ethephon, an ethylene-releasing compound, was mitigated by exogenously applied GSH in sweet potato [22,23]. Furthermore, ethylene signalling was involved in the stimulation of GSH synthesis in aluminium (Al)-exposed *A. thaliana* plants [24].

Based on these data, it can be stated that ethylene and GSH are closely intertwined. Nevertheless, a better understanding of this reciprocity is required to further unravel the role of both key determinants in the short-term responses to Cd exposure. A previous study by Deckers et al. (2020) described the spatiotemporal sequence of events concerning different pressure points under Cd stress, and the depletion of root GSH levels was brought forward as a determinant of the initial alarm phase [10]. This alert response was suggested to be crucial to provoke an optimal response under Cd stress and would later on define the new steady state, termed acclimation. Therefore, we focussed on how a diminished GSH synthesis would affect this early Cd-induced alert response and how this alters stabilisation, which provides a first indication of acclimation. The transcriptional and metabolic responses related to the oxidative challenge and ethylene were studied in both wild-type and GSH-deficient *cad2-1* mutant plants. Three-week-old plants were exposed to 5 µM Cd in a short-term (2 h, 24 h, 72 h) exposure set-up, and Cd-induced root and leaf responses were compared between both genotypes.

## 2. Materials and Methods

### 2.1. Plant Cultivation, Cadmium Exposure, and Sampling

Seeds of both WT and Cd-sensitive *cad2-1* mutant *Arabidopsis thaliana* plants (Columbia background) were surface-sterilised and incubated in darkness for 3 days at 4 °C. The *cad2-1* mutant (deletion of P237, K238, and V239L) seeds were kindly provided by Prof. Dr Christopher Cobbett (Melbourne University, Australia) [11]. Seedlings were hydroponically grown using a modified Hoagland nutrient solution and with purified sand as a substrate [25,26]. Plants were grown under a relative humidity of 65% and a photoperiod of 12 h, with a day temperature of 22 °C and a night temperature of 18 °C. The photosynthetically active radiation (PAR) of sunlight was simulated using a combination of red, far-red, and blue light (Philips Green-Power LED modules) providing a photosynthetic photon flux density of 170 µmol m^−2^ s^−1^ at the rosette level. After a 3-week cultivation period under control conditions, both WT and mutant plants were exposed to 0 µM Cd (control) or 5 µM Cd via administration of CdSO_4_ to the nutrient solution. After 2 h, 24 h, or 72 h of exposure, roots and complete rosettes were separately harvested. The fresh weight was determined, and samples were snap-frozen using liquid nitrogen and stored at −70 °C, unless mentioned otherwise.

### 2.2. Determination of Cadmium Concentrations in Roots and Leaves

During harvest, roots were incubated in 10 mM Pb(NO_3_)_2_ for 15 min at 4 °C, to exchange surface-bound metals, and subsequently rinsed in distilled water. Harvested leaves were rinsed with distilled water. Next, samples were dried in an oven at 80 °C, weighed, and digested in HNO_3_ (70–71%) and HCl (37%) using a heat block. Prior to analysis, extracts were dissolved in a final solution of 2% HCl. Using inductively coupled plasma optical emission spectrometry (ICP-OES; Agilent Technologies 700 Series, Santa Clara, CA, USA), Cd concentrations were quantified. Standard (trace elements in cabbage powder, No. 679, Standard Reference Material) samples were included for reference purpose.

### 2.3. Gene Expression Analysis

To investigate transcriptional responses under Cd exposure, gene expression analysis was performed. Frozen root and leaf samples were homogenised using two stainless steel beads and the Retsch Mixer Mill MM400 (Retsch, Haan, Germany). First, RNA extraction was performed using the RNAqueous^®^ Kit (Thermo Fisher Scientific, Waltman, MA, USA) according to the manufacturer’s instructions. Sample purity and RNA concentrations were spectrophotometrically determined using the Nanodrop^®^ ND-1000 spectrophometer (Thermo Fisher Scientific, Waltman, MA, USA) and samples were stored at −70 °C. Following RNA isolation, cDNA synthesis was conducted using equal amounts of RNA as input (1 µg) for the reverse transcription reaction, using the Primescript^TM^ RT reagent Kit (Takara Bio Inc., Kusatsu, Japan). Residual genomic DNA was removed prior to cDNA synthesis by pre-treating the samples with DNase using the TURBO DNA-*free*^TM^ Kit (Thermo Fisher Scientific, Waltman, MA, USA). The cDNA samples were ten-fold diluted using a 1/10 Tris-EDTA buffer (1 mM Tris-HCl, 0.1 mM Na_2_-EDTA, pH 8.0) and stored at −20 °C. To determine the expression levels of the reference genes and genes of interest (GOIs; Supplementary Tables S1 and S2), quantitative real-time PCR (qPCR) was performed using the QuantStudio™ 3 Real-Time PCR System (Applied Biosystems, Thermo Fisher Scientific, Foster City, CA, USA) and the Quantinova^TM^ SYBR^®^ Green PCR Kit (Qiagen, Hilden, Germany). Universal cycling conditions (2 min at 95 °C, 40 cycles of 5 s at 95 °C, and 12 s at 60 °C) were used for amplification. Subsequently, amplicon specificity was verified by the generation of a melting curve. Reactions contained 2 µL of the diluted cDNA template, 0.3 µL of forward and reverse primers (300 nM unless mentioned otherwise in Table S1), 5 µL Quantinova^TM^ SYBR^®^ Green Dye, 0.05 µL ROX reference dye, and 2.35 µL RNase-free H_2_O in a total reaction volume of 10 µL. Relative gene expression levels were calculated from the obtained Cq values using the 2^−ΔCq^ method. Data were normalised to correct for technical variation using the geometric mean of at least three reference genes (Table S2). The latter were selected from 10 candidate reference genes using the GrayNorm Algorithm [27]. Gene-specific primers were designed using Primer 3, and the primer specificity was checked in silico using BLAST (http://www.arabidopsis.org/Blast/index.jsp accessed on 1 November 2021). An optimal reaction efficiency was guaranteed, as primer efficiencies were evaluated based on a two-fold dilution series generated from a pooled sample that served as standard curve. Only primers with an efficiency between 90% and 110% were used. The qPCR parameters, defined by the Minimum Information for publication of Quantitative real-time PCR Experiments (MIQE) guidelines, are presented in Supplementary Table S3 [28].

### 2.4. Determination of Glutathione Concentrations 

Concentrations of GSH in both roots and leaves were spectrophotometrically determined using the plater reader method according to Jozefczak et al. (2014) [5]. Frozen samples were shredded using the Retsch Mixer Mill MM400 (Retsch, Haan, Germany) and two stainless steel beads. Samples were vortexed together with 200 mM HCl (6.66 mL mg^−1^ leaf fresh weight; 9.4 mL mg^−1^ root fresh weight) to prevent oxidation of the sample. After centrifugation (10 min, 16,000× *g*, 4 °C), the pH of the samples was adjusted to 4.5 using 200 mM NaOH, while keeping the samples at 4 °C. The GSH-dependent reduction of 5,5-dithiobis (2-nitro-benzoic acid) (DTNB, 600 µM) was monitored at 412 nm using a FLUOstar Omega microplate reader (BMG Labtech, Ortenberg, Germany). Oxidised GSH (GSSG) present in the sample or formed during the reduction of DTNB was reduced by glutathione reductase (1 U mL^−1^) using NADPH (500 mM) as an electron donor. The change in absorbance over time was proportional to the total GSH concentration in the sample, which was determined using a GSH standard curve. To determine the amount of GSSG, the samples were incubated with 2-vinyl-pyridine (2-VP), which functions as a masking agent for GSH (2-VP, 1% *v*/*v*). Samples were incubated for 30 min at room temperature. Complexed GSH and 2-VP were precipitated by centrifugation (10 min, 16,000× *g*, 4 °C), and the obtained supernatant was centrifuged again (10 min, 16,000× *g*, 4 °C) to remove all reduced GSH and 2-VP prior to the plate reader measurement.

### 2.5. Hydrogen Peroxide Measurements

Using the Amplex^TM^ Red Hydrogen Peroxide/Peroxidase Assay Kit (Invitrogen, Thermo Fisher Scientific, Carlsbad, CA, USA), relative hydrogen peroxide (H_2_O_2_) concentrations were determined in roots and leaves. Frozen samples were shredded using two stainless steel beads and the Retsch Mixer Mill MM 400 (Retsch, Haan, Germany). Samples were extracted in 500 µL 1× reaction buffer and incubated at room temperature for 30 min while being continuously shaken. To precipitate plant debris, samples were centrifuged at 12,000× *g* for 5 min. Using a 96-well plate, reactions were performed in a total reaction volume of 100 µL containing 95 µL working solution, consisting of 100 µM Amplex^TM^ Red and 0.2 U mL^−1^ horseradish peroxidase, to which 5 µL sample was added. Prior to the measurement, the 96-well plate was incubated in the dark at 30 °C for 2 h. An excitation wavelength of 560 nm was used and resurofin fluorescence was monitored at 590 nm in a FLUOstar^®^ Omega microplate reader (BMG Labtech, Ortenberg, Germany).

### 2.6. Determination of Free ACC and Conjugated ACC Concentrations

Root and leaf samples (approx. 100 mg fresh material) were homogenised under frozen conditions using two stainless steel beads and the Retsch Mixer Mill MM 400 (Retsch, Haan, Germany). During extraction in 400 µL 80 v% ice-cold methanol, [^2^H_4_] ACC (600 pmol, Sigma, St. Louis, MO, USA) was added as internal standard to allow quantification, and half a milligram of OASIS HLB 0.3 µm solid phase bulk packing material (WATERS, Milford, MA, USA) was added to bind pigments. After centrifugation (20,817× g, 15 min, 4 °C), half of the extract was used to analyse free ACC using ES(+)UPLC-MS/MS (ACQUITY TQD, WATERS) in combination with a Waters column ACQUITY UPLC BEH Amide 1.7 µm Column. Solvent A: 0.1% FA in water, solvent B: 0.1% FA in acetonitrile; flow 0.4 mL/min, gradient 0–2 min: 15.0% A/85.0% B, 2–5.8 min: linear gradient to 35.0%/65.0% B, 5.8–6.4 min: linear gradient to 80.0% A/20.0% B, isocratic at 80.0% A/20.0% B until 7 min. Injection Mode: partial loop, injection volume 6 µL. Specific transitions selected for MRM (dwell time 0.034 sec. for each transition): 102.0 > 56.0 (cone: 22, collision 16.0) and 102.0 > 84.0 (cone 14.0, collision 8.0) for ACC, 106.0 > 60.0 (cone: 22.0, collision energy 16.0), and 106.10 > 88.00 (cone 14.0, collision 8.0) for d4-ACC. The total amount of conjugated ACC was analysed after acid hydrolysis of the second half of the extract in 2 M HCl at 100 °C during 2 h. After drying under a nitrogen stream (TurboVap II, Zymark, MA, USA), the samples were dissolved in 100 µL 80 v% MeOH and analysed as ACC following the procedure described above.

### 2.7. Statistical Analysis

Firstly, outliers were identified using the Extreme Studentised Deviate method (GraphPad Software, La Jolla, CA, USA) with the significance level set at 0.05. All subsequent statistical analyses were performed in R version 3.3.1 (R Foundation for Statistical Computing, 2016, Vienna, Austria). Normality and homoscedasticity of the data were checked using the Shapiro–Wilk and Barlett’s test, respectively. If these assumptions were not met, data were transformed (logarithm, square root, inverse, exponent), and gene expression data were standardly log-transformed. In case both assumptions were met, a Student’s *t*-test or 2-way ANOVA were used at significance level 0.05. The 2-way ANOVA was followed by a post-hoc Tukey–Kramer test for multiple comparisons. However, if normality and homoscedasticity were not obtained by transformation, a non-parametric Kruskal–Wallis test was used, followed by Wilcoxon Signed Rank sum test for pairwise comparison of the data.

## 3. Results

As demonstrated by Deckers et al. (2020), the Cd-induced stress response follows the typical stress response curve and consist of an alarming phase, a restitution phase, and eventually, acclimation [10]. Nevertheless, if acclimatory responses are too weak, this will lead to the degradation of the system [29]. Furthermore, this response was proposed to be largely defined by GSH, and especially by the early Cd-induced depletion at the root level that serves as an alarm phase [10]. To elucidate how diminished GSH synthesis alters Cd-induced stress response, hydroponically grown WT and *cad2-1* mutant *A. thaliana* plants were either grown under control conditions or exposed to 5 µM Cd. This environmentally relevant Cd concentration was found in soil pore water in polluted areas and was demonstrated to be sublethal to WT plants, as it allowed completion of the plant’s life cycle [26,30]. Plants were harvested at three different time points, namely 2 h, 24 h, and 72 h. The first two were previously shown to encompass major signalling events, while Cd-induced plant responses are known to stabilise after 72 h, when a new steady state is reached, revealing a first indication of acclimation [5,10].

### 3.1. Growth Responses and Cadmium Concentrations

**Figure 1:**
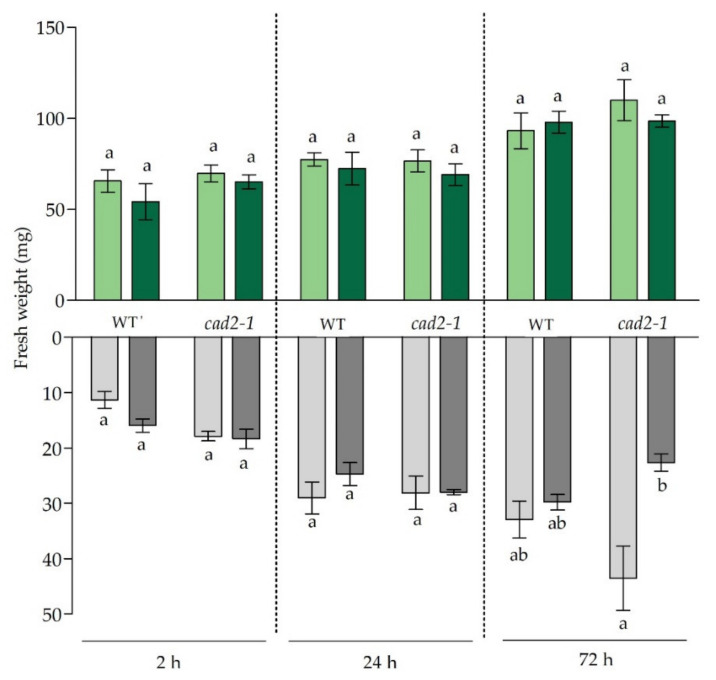
Rosette (green bars) and root (grey bars) fresh weight (mg) of 3-week-old wild-type (WT) and *cad2-1* mutant Arabidopsis thaliana plants grown under control conditions (0 µM CdSO_4_, light bars) or exposed to 5 µM CdSO_4_ (dark bars) during 2 h, 24 h, and 72 h. Data are presented as the mean ± S.E. of at least eight biological independent replicates. Significant differences (2-way ANOVA: *p* < 0.05) within each time point and organ are indicated with different letters.

Under control conditions, no significant differences were observed between fresh weights of roots and leaves of both genotypes. While no Cd-induced growth effects were observed in the leaves, the root fresh weight was negatively impacted after 72 h in mutant plants, which was not observed for WT plants (Figure 1). In accordance, previous studies observed no leaf growth effects after 24 h and 72 h of Cd exposure for both genotypes [31,32].

For both genotypes, Cd concentrations were higher in roots compared to leaves at the three considered time points. At the early time point (2 h), Cd concentrations in the leaves of mutant plants were significantly higher compared to the WT plants (Table 1). Hence, the ratio of leaf-to-root Cd concentrations, termed the translocation factor, was significantly higher in the GSH-deficient mutant as compared to the WT after 2 h. However, after 24 h and 72 h of exposure, the opposite was observed, and leaf Cd concentrations, as well as translocation, were significantly lower in mutant plants (Table 1). At these later time points, the translocation factor was calculated to be around 50% in WT plants, meaning that almost one-third of the Cd taken up by the roots was translocated to the leaves. In mutant plants, Cd translocation was strongly limited.

### 3.2. The Dual Role of Glutathione as Antioxidant and Chelator under Glutathione-Deficient Conditions

**Figure 2:**
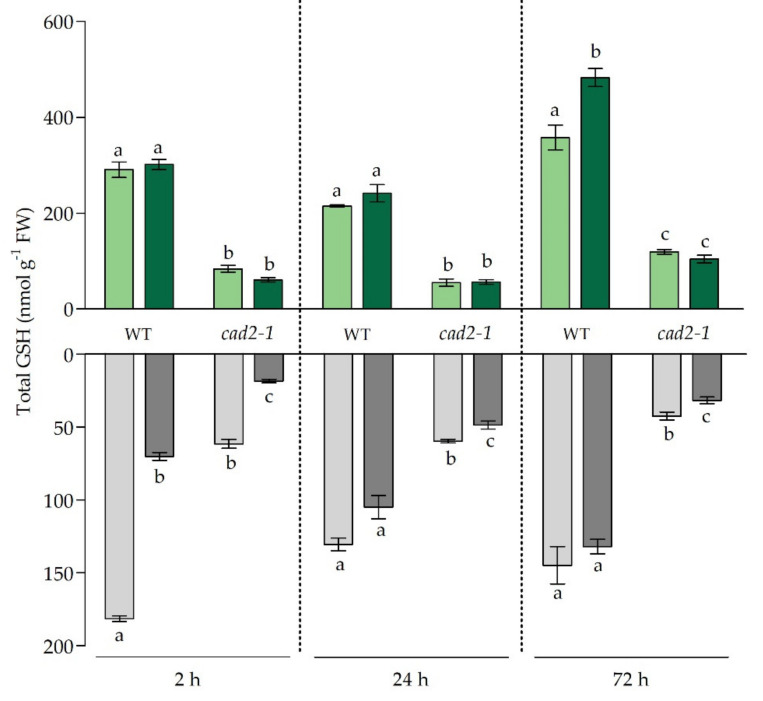
Total glutathione (GSH) concentrations (nmol g^−1^ fresh weight) in leaves (green bars) and roots (grey bars) of 3-week-old wild-type (WT) and *cad2-1* Arabidopsis thaliana plants grown under control conditions (0 µM CdSO_4_, light bars) or exposed to 5 µM CdSO_4_ (dark bars) during 2 h, 24 h, and 72 h. Data are presented as the mean ± S.E. of at least three biological independent replicates. Significant differences (2-way ANOVA: *p* < 0.05) within each time point and organ are indicated with different letters.

Because of a mutation in the *GSH1* gene, which results in a deletion close to the active site of the γ-glutamate-cysteine ligase enzyme, the first and rate-limiting step of GSH synthesis is less effective in the *cad2-1* mutant. Hence, this mutant contains only 30–45% of the WT GSH concentrations in its cells [11,12]. This was confirmed by our data as control GSH concentrations were significantly lower in the roots and leaves of mutant plants (Figure 2). After 2 h of Cd exposure, root GSH concentrations became strongly depleted to a similar extent in both genotypes. More precisely, GSH concentrations were only 39% and 30% of the control concentrations in WT and mutant plants, respectively. After 24 h, root GSH concentrations were restored to control concentrations in Cd-exposed WT plants, whereas in mutant plants, the GSH concentrations remained significantly lowered under Cd exposure (Figure 2). Similar results were observed after 72 h, as GSH concentrations in mutant roots remained significantly lowered, while this was not the case for Cd-exposed WT plants. At the leaf level, no GSH depletion was observed after 2 h of exposure, nor at later time points when leaf Cd concentrations reached similar levels as observed after 2 h in roots of WT plants. To the contrary, after 72 h, leaf GSH levels were significantly enhanced in WT plants under Cd exposure, but this was not observed in *cad2-1* mutant plants (Figure 2). 

In roots of the *cad2-1* mutant, the GSH depletion coincided with a significant induction of all three considered GSH metabolism genes (*GSH1*, *GSH2*, and *GR1*) measured. Furthermore, the transcriptional activator of *GSH1*, *ZAT6*, was also significantly induced early on during the stress response and, notably, to a much larger extent in mutant plants than in WT plants (Table 2). After 24 h, even though GSH metabolism genes were significantly induced in roots of both genotypes, the GSH levels were recovered to control levels only in the WT plants (Figure 2). These pronounced transcriptional inductions vanished after 72 h. In the leaves, clear differences in responses at the transcript level were observed after 24 h. More specifically, while in WT plants all three GSH metabolism genes were significantly induced, in mutants this was limited to *GSH2*. In WT plants this transcriptional induction of GSH synthesis genes preceded the enhanced GSH concentration observed in leaves after 72 h (Table 2 and Figure 2).

### 3.3. Balancing the Cadmium-Induced Oxidative Challenge under Glutathione-Limiting Conditions

**Figure 3:**
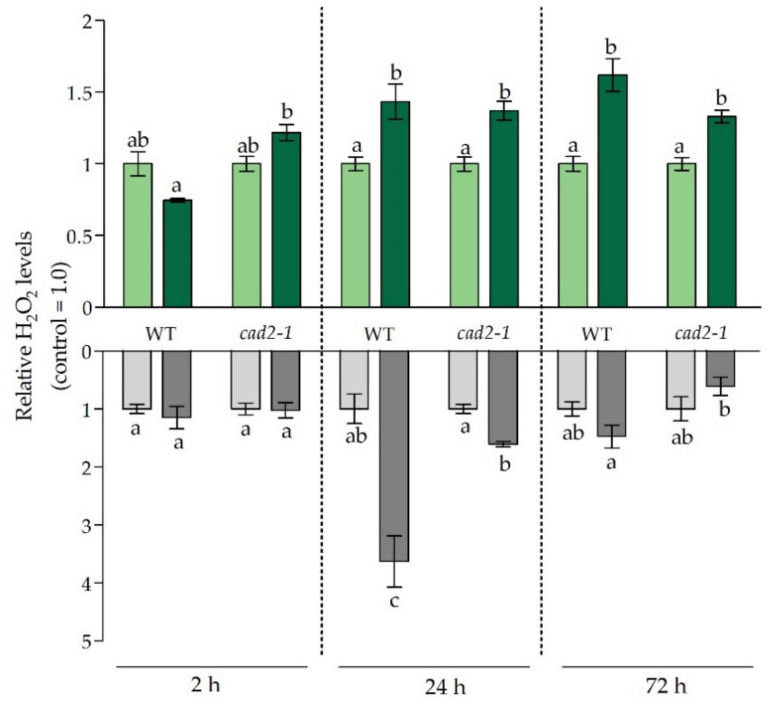
Relative hydrogen peroxide (H_2_O_2_) levels in leaves (green bars) and roots (grey bars) of 3-week-old wild-type (WT) and *cad2-1* mutant Arabidopsis thaliana plants grown under control conditions (0 µM CdSO_4_, light bars) or exposed to 5 µM CdSO_4_ (dark bars) during 2 h, 24 h, and 72 h. Data are presented as the mean ± S.E. of at least three biological independent replicates relative to the control of the corresponding genotype set at 1.00. Significant differences (2-way ANOVA: *p* < 0.05) within each time point are indicated with different letters.

Cadmium exposure generally leads to increased generation of ROS, tipping the balance between antioxidants and pro-oxidants towards the latter. Although ROS were, for a long time, considered damaging compounds, nowadays it is clear that their function is more nuanced, as they are involved in signalling. Hydrogen peroxide (H_2_O_2_), in particular, is often put forward as important signalling molecule due to its longevity, relatively limited toxicity, and ability to cross membranes [33]. Therefore, we focused on the spatiotemporal profile of H_2_O_2_ production in both organs of plants under short-term Cd exposure (Figure 3).

**Figure 4:**
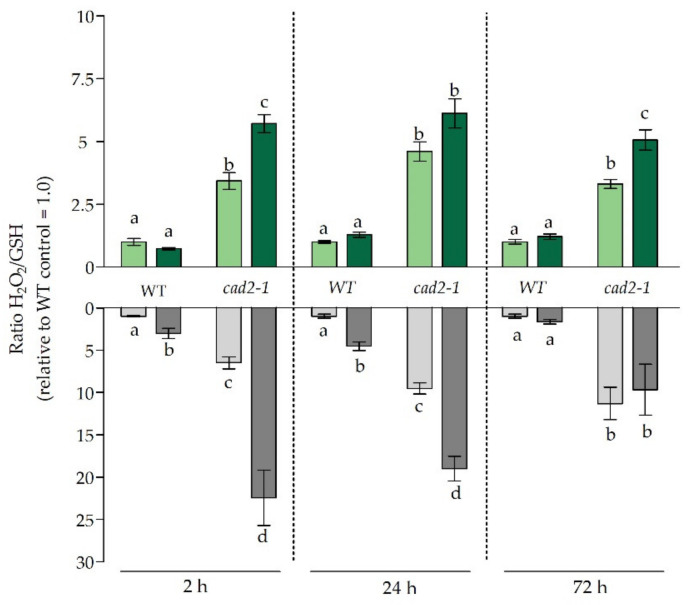
Relative H_2_O_2_/GSH ratio in leaves (green bars) and roots (grey bars) of 3-week-old wild-type (WT) and *cad2-1* Arabidopsis thaliana plants grown under control conditions (0 µM CdSO_4_, light bars) or exposed to 5 µM CdSO_4_ (dark bars) during 2 h, 24 h, and 72 h. Data are presented as the mean ± S.E. of at least three biological independent replicates relative to the control of the WT set at 1.00 set. Significant differences (2-way ANOVA: *p* < 0.05) within each time point are indicated with different letters.

Additionally, as H_2_O_2_ and GSH often act in concert and are both key determinants of the plant’s redox status, the ratio between relative H_2_O_2_ levels and GSH concentrations was determined (Figure 4). This provided a way to obtain an integrated view on the Cd-induced redox changes in both roots and leaves of WT and mutant plants. Under control conditions, this ratio was significantly more elevated in both organs of non-exposed mutant plants as compared to WT plants (Figure 4). This was attributable to the higher basal H_2_O_2_ levels observed in roots of the *cad2-1* mutant (Figure S1) and, at the same time, lower GSH levels in mutant roots as compared to WT roots (Figure 2). This coincided with significantly higher transcript levels of a subset of oxidative stress marker genes, previously identified by Gadjev et al. (2006), in roots of non-exposed mutant plants (Figure S2) [34]. As control H_2_O_2_ levels were significantly higher in the roots of *cad2-1* mutant plants, the Cd-induced stimulation of H_2_O_2_ production observed in WT plants after 24 h was significantly less pronounced in mutant plants (Figure 3). Overall, regarding H_2_O_2_, Cd-induced leaf responses were similar for both WT and mutant plants. In the early phase of the Cd-induced stress responses, the redox ratio became significantly elevated in the roots and, more specifically, to a similar extent in both genotypes (Figure 4). This significant increase was also observed after 24 h, while it vanished after 72 h. In leaves, significant increases were only observed for mutant plants, and more precisely, after 2 h and 72 h.

When considering root transcript levels of the oxidative-challenge-related genes, an overall stronger induction was observed in the mutant plants after 2 h of exposure in comparison to the WT plants (Table 3). After 24 h, these genes were induced in both genotypes and the extent of induction varied strongly. Similar to the root responses after 2 h, the transcript levels, in general, became significantly more enhanced after 72 h in mutant roots. Only the oxidative stress marker *AT2G43510* became induced to a similar and very large extent in both genotypes (Table 3). In the leaves, the oxidative stress markers were significantly induced in WT plants upon 24 h of exposure and not, or only to a limited extent, in mutant plants (Table 3). After 72 h of exposure, these transcriptional inductions mainly faded. In addition, the expression levels of ROS-producing NADPH oxidases as well as ROS-scavenging genes were determined (Tables S5 and S6). In general, the transcriptional responses of ROS-generating NADPH oxidases in the roots were similar for both genotypes (Table S6). Both *RBOHD* and *RBOHF* were significantly induced after Cd exposure, and transcript levels remained significantly elevated up to 72 h. In the roots, however, the *RBOHC* gene did not show a significant induction. The gene expression of the antioxidative enzyme ASCORBATE PEROXIDASE 2 (APX2) was significantly more increased in the roots of Cd-exposed mutant plants compared to WT plants throughout the exposure time frame. The upregulation of *CAT1* was only significantly higher in mutant plants compared to WT plants after 72 h (Table S5). In addition, *APX1* was significantly induced in the mutant after 24 h of exposure. In the leaves, the low abundant *RBOHC* gene was significantly induced upon 24 h of Cd exposure in WT plants. More specifically, the transcript levels were increased almost 80-fold in WT plants, while no increase was observed in mutant plants (Table S6). Likewise, the expression levels of *RBOHD* and *RBOHF* were also significantly increased after 24 h in WT plants, while no enhanced transcript levels were observed for the mutant plants. Concerning the transcript levels of the antioxidative enzymes, inductions were only observed for WT plants, and this after 24 h and 72 h. For *CAT2*, a significant decrease in transcript levels was detected in the leaves of WT plants after 24 h of exposure to Cd. For mutant plants only, decreased gene expression levels were observed, and this for *APX1* and *APX2* after 2 h and 72 h, respectively (Table S5).

### 3.4. The Effect of Diminished Glutathione Synthesis on Ethylene-Related Responses under Cadmium Stress

**Figure 5:**
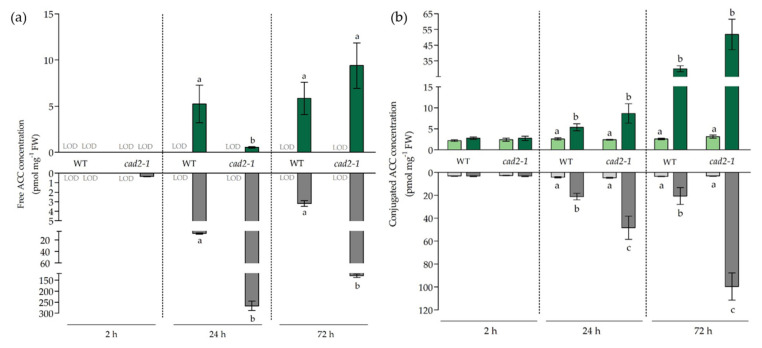
(**a**) Free and (**b**) conjugated 1-aminocyclopropane-1-carboxylic acid (ACC and ACCc) concentrations (pmol mg-1 fresh weight) in leaves and roots of 3-week-old wild-type (WT) and *cad2-1* mutant Arabidopsis thaliana plants grown under control conditions (0 µM CdSO_4_, light bars) or exposed to 5 µM CdSO_4_ (dark bars) during 2 h, 24 h, and 72 h. Data are presented as the mean ± S.E. of at least four biological independent replicates. Significant differences (2-way ANOVA: *p* < 0.05) within each time point are indicated with different letters. L.O.D.: limit of detection.

Schellingen et al. (2015) demonstrated that ethylene signalling is required for the stimulation of GSH metabolism in leaves of Cd-exposed *A. thaliana* plants [21]. In our study, the effects of impaired GSH synthesis on the transcript levels of ethylene biosynthesis and responsive genes were investigated together with the concentrations of free ACC as well as conjugated ACC (ACCc) (Table 4 and Figure 5). This direct precursor of ethylene is synthesised from SAM, which is, under non-stressed conditions, considered to be the rate-limiting step. In addition, the conjugation of free ACC negatively affects the production of ethylene by limiting the amount of ACC available for conversion by ACO [16]. In this study, the ethylene-related responses were more pronounced in the roots compared to the leaves (Table 4). The *ACS6* gene was significantly induced early after the start of exposure to Cd in roots of both genotypes, but to a significantly larger extent in the *cad2-1* mutant. Transcript levels of this gene remained significantly enhanced at later time points and were again induced to a significantly larger extent in mutant plants compared to WT plants after 72 h. The second Cd-responsive ACC synthase gene, *ACS2*, was significantly induced after 24 h and 72 h, and at both time points the transcript levels were significantly higher in *cad2-1* mutant roots compared to WT roots (Table 4). For both *ACO* genes that are known to be involved in Cd-induced ethylene biosynthesis, responses were similar between roots of both genotypes. While *ACO2* was only significantly induced upon 24 h of exposure, *ACO4* was transcriptionally upregulated at all three time points. Similar to the transcriptional profile of *ACS6*, the ethylene-responsive *ERF1* gene was significantly induced in both genotypes throughout the exposure time frame, and a significantly larger upregulation was observed in the roots of mutant as compared to WT plants after 2 h and 72 h. After 24 h of exposure, the opposite was true. The upstream regulators involved in the Cd-induced transcriptional activation of *ACS2* and *ACS6* were also upregulated in roots after Cd exposure. More specifically, *OXI1*, *MPK3,* and *WRKY33* were significantly induced throughout the entire exposure time frame. When differences in the extent of induction were observed, they were significantly higher in roots of the mutant plants compared to WT plants. For *MPK6*, the Cd-induced transcriptional effects were limited to 2 h and 24 h of exposure, and while transcript levels were only significantly enhanced in WT plants upon 24 h of exposure, this induction could be observed already after 2 h in mutant plants. Likewise, free ACC concentrations were significantly increased early on in the roots of mutants and remained significantly more elevated compared to WT levels after 24 h and 72 h of exposure (Figure 5). The production of ACCc was significantly increased after 24 h and 72 h and, again, significantly higher in roots of mutant plants than in WT plants.

In leaves, transcriptional responses were generally delayed as compared to roots. Only *ACS6* was already significantly induced after 2 h of Cd exposure in mutant leaves. After 24 h, transcript levels of ethylene-related genes peaked significantly for WT plants, while inductions were absent or significantly lower in mutant leaves (Table 4). In general, the ethylene response seemed less pronounced in the leaves of mutant plants after 24 h compared to WT plants. After 72 h, most genes were no longer transcriptionally induced or to a much smaller extent for both genotypes. Similarly, compared to WT plants, ACC concentrations were significantly lower in the leaves of the *cad2-1* mutant after 24 h of exposure. Nevertheless, a tendency towards higher ACC concentrations was observed after 72 h in mutant leaves. After 24 h and 72 h, the ACCc concentrations were also significantly higher in Cd-exposed plants in general. After 72 h, similar to the ACC concentrations, a tendency towards higher ACC conjugation was observed for the leaves of mutant plants compared to WT plants (Figure 5).

## 4. Discussion

As a major antioxidant and Cd chelator, GSH can be considered a key determinant of the Cd-induced stress response. More specifically, it was shown that the early Cd-induced stress response was largely defined by a steep decrease in root GSH concentrations in *A. thaliana* [5,10]. This early depletion is caused by the allocation of GSH to the production of Cd-chelating PCs, thereby restricting its availability for antioxidative defence [5]. Nevertheless, rather than being merely an adverse effect, it was suggested by Deckers et al. (2020) that the GSH depletion serves as an alarm response and mediates stress signalling, which is required for optimal acclimation later on [10]. In the current study, the GSH-deficient *cad2-1* mutant was used to further elucidate how impaired GSH synthesis impacts the short-term responses and, more specifically, the signalling responses under acute Cd exposure, which are initiated by the early depletion of root GSH concentrations. For this purpose, three different time points were considered that encompass signalling (2 h and 24 h) and stabilisation (72 h). The latter provides a first indication of the new homeostasis in function of acclimation. 

As indicated by Deckers et al. (2020), Cd-induced responses differ between roots and leaves of *A. thaliana* in terms of timing and severity; therefore, both organs were studied separately [10]. While it was previously shown that the *cad2-1* mutant has similar GSH1 protein levels as WT *A. thaliana* plants, a deletion close to its active site impairs its enzymatic activity, resulting in only 30–45% of WT GSH concentrations [12,35]. Our data showed that, although *cad2-1* mutants are limited in available GSH, the rapid depletion of root GSH concentrations still occurred after 2 h of Cd exposure (Figure 2). This indicates that, during the early phase of the stress response, the chelating capacity of GSH is prioritised over its antioxidative function even under GSH-limiting conditions. Concomitantly, the constraints posed on the antioxidative capacity of GSH at this early time point became apparent at the transcript level by the significantly higher induction of oxidative challenge-related genes in roots of mutant plants (Table 3)**.** Because alterations of GSH concentrations were previously demonstrated to be important modulators of the stress-induced increases in H_2_O_2_, the ratio between oxidising H_2_O_2_ and GSH was considered and provided us with an integrated view on the Cd-induced redox changes [10,35,36]. In accordance with the oxidative-challenge-related transcripts, this ratio was significantly more increased in roots of the *cad2-1* mutant after 2 h of Cd exposure in comparison to WT plants (Figure 4). This indicates that the redox ratio (H_2_O_2_/GSH), which was already elevated in the mutant plants under control conditions, became even more oxidised because of the Cd-induced depletion of GSH. Moreover, it was demonstrated by Han et al. (2013) that in plants the redox status is controlled in such a way that GSH depletion inhibits GSH oxidation, and strong alterations in GSH concentrations are sufficient to alter the cell’s redox potential and stimulate GSH synthesis [36]. Likewise, Deckers et al. (2020) demonstrated that in WT *A. thaliana* plants, the early Cd-induced root GSH depletion did not lead to increased oxidation of GSH [10]. This was also observed in our study, as the percentage of reduced GSH remained tightly controlled above 90% (Table S4). Furthermore, *GR1* was significantly induced early on in the roots of mutant plants only, hinting towards an increased turnover of GSSG to GSH, an enhanced H_2_O_2_ metabolism, and signalling at this early time point in mutant roots (Table 2) [37].

As both GSH synthesis genes, *GSH1* and *GSH2*, were already upregulated after 2 h of exposure in the roots of mutant plants (Table 2), it could be suggested that the Cd-induced redox changes, which were more pronounced in the *cad2-1* mutant, possibly serve as an early driving force to stimulate GSH synthesis. This can be considered an accelerated response, as GSH metabolism genes were only stimulated after 24 h in roots of WT plants (Table 2). More precisely, Deckers et al. (2020) indicated that the induction of *GSH1* and *GSH2* occurred only after 6 h of Cd exposure in roots of WT plants [10]. The enhanced GSH production is further confirmed by the elevation in GSH concentrations after 24 h in both genotypes. Note, however, that while in WT plants the GSH concentrations were restored to control levels, in mutant roots these concentrations remained significantly lower (Figure 2). Furthermore, the zinc finger transcription factor, *ZAT6*, became induced to a significantly larger extent in roots of the *cad2-1* mutant upon Cd exposure (Table 2). This transcription factor was demonstrated to be important for Cd tolerance by functioning as a direct and transcriptional activator of *GSH1* [38]. Correspondingly, Chen et al. (2016) showed that the *zat6* mutant displayed a similar phenotype to the *cad2-1* mutant under Cd exposure [38]. In our study, the early and significantly stronger upregulation of *ZAT6* in the mutant coincided with the transcriptional upregulation of *MPK6* after 2 h (Table 4). Several studies already demonstrated that the activity, as well as gene expression, of MAPK6 are strongly induced upon Cd exposure and depend on ROS accumulation [18,19,39,40]. This kinase, in its turn, is known to phosphorylate and activate ZAT6 [39]. Therefore, we suggest that the more severe oxidative challenge in the mutant roots could result in the upregulation and activation of MPK6, which in its turn would phosphorylate ZAT6, leading to increased ZAT6 activation and possibly a higher transcript demand as observed in our study. In addition, the strongly increased expression of *ZAT6* coincided with the significant induction of *GSH1*, which did not occur in WT roots (Table 2). This further supports the accelerated ZAT6-mediated induction of *GSH1* in *cad2-1* mutant plants as an attempt to rapidly salvage the low GSH levels.

Another zinc finger protein, ZAT12, which is implemented in oxidative stress and responsive to H_2_O_2_, was transcriptionally induced early on during the exposure time frame and, again, to a significantly larger extent in mutant plants (Table 2). The importance of ZAT12 in the oxidative stress response, evoked by paraquat or H_2_O_2_ application, was illustrated by the lack of, amongst others, *APX1* induction in *zat12* mutants [40]. Moreover, its expression levels increased with increasing H_2_O_2_ levels in the *apx1* loss-of-function mutant [41]. Likewise, Jozefczak (2014) demonstrated that *APX1*, along with the transcripts of other antioxidants, became significantly induced in roots of *cad2-1* mutant plants after 24 h of Cd exposure, while this induction was absent in roots of WT plants [32]. In our study, *APX1* and *APX2,* in particular, were significantly more strongly upregulated in roots of exposed mutant plants compared to WT plants (Table S5). These observations hint towards the activation of alternative antioxidative pathways when GSH concentrations are inadequate.

Besides GSH, the stress hormone ethylene is often implemented in the responses to Cd stress and the crosstalk between these two key regulators in the responses to (a)biotic stress is well documented [21,22,23,42,43,44]. While Datta et al. (2015) suggested a positive effect of *GSH1* overexpression, and hence increased GSH concentrations, on the transcription of *ACS2* and *ACS6*, we observed that decreased GSH synthesis as well as a further depletion after 2 h of Cd exposure led to an enhanced *ACS6* expression and elevated ACC concentrations in the roots of mutant plants (Table 4 and Figure 5) [43]. At first sight, this contrasts the findings by Datta et al. (2015). However, they demonstrated under non-stressed conditions a positive and direct effect of endogenously increased GSH on WKRY33, which was independent of MPKs [43]. In our study, a strong induction of *MPK3* and *MPK6* was observed that coincided with the induction of *WRKY33* in the roots. Therefore, we suggest that during the short-term Cd-induced stress response, the upregulation of *ACS2* and *ACS6* does not directly depend on GSH concentrations. More specifically, we propose that the more pronounced oxidative challenge, due to depletion of already limited GSH concentrations, was perceived by the oxidative stress-inducible kinase OXI1, of which the transcript levels were also upregulated early on during the Cd-induced stress response (Table 4). As demonstrated before, induction of OXI1 could lead to the transcriptional activation of the MPK pathway, which targets WKRY33, leading to increased expression of *ACS6* and later on also *ACS2* [17,45,46,47,48]. While both ACS isoforms are known to be largely responsible for enhanced ethylene synthesis in responses to a plethora of stresses, our data demonstrated that the *ACS6* gene acts early in the Cd-induced response (Table 4) [17,45,48]. Moreover, the significantly stronger induction of *ACS6* after 2 h followed by the more pronounced upregulation of *ACS2* corresponded to the significantly higher root ACC levels observed in mutant plants compared to WT plants (Figure 5). While this resulted in accelerated ethylene signalling after 2 h of exposure, this did not enhance the ethylene response (i.e., the expression of *ERF1*) after 24 h in comparison to WT plants (Table 4). To the contrary, the observed transcriptional upregulations were significantly lower after 24 h in roots of mutant plants as compared to WT plants (Table 4). Based on these findings, it could be suggested that increased ACC concentrations are caused by a stronger upregulation of ACC synthesis but also by diminished ethylene production.

It is known that ethylene production and signalling are required for ROS production and, concomitantly, induction of appropriate stress responses [49]. In accordance, the peak in H_2_O_2_ production coincided with the strong induction of ethylene-related genes in WT plants and was significantly lower in mutant plants (Figure 3 and Table 4). In general, it should be noted that the H_2_O_2_ levels and the H_2_O_2_/GSH ratio were significantly elevated under control conditions for roots of mutant plants (Figure 4 and Figure S1). As mutant plants are naturally more challenged, it is possible that the stress response is more confined in order to avoid further damage and cell death. Moreover, we suggest that a more rapid and severe stress response after 2 h at the root level accelerates signalling in mutant plants and is counteracted after 24 h in order to prevent adverse effects. However, as stress persists, and because of the inability to restore GSH levels, it seems that stabilisation, which is typically observed in WT plants after 72 h and provides a first indication of acclimation, does not occur or is at least delayed in mutants. More precisely, in mutant roots the majority of ethylene-related genes were still upregulated to a significantly larger extent compared to WT plants (Table 4), oxidative stress was more pronounced at the transcript level compared to WT plants (Table 3), and ACC levels remained strongly elevated after 72 h of exposure (Figure 5a). However, as a significantly higher concentration of ACCc was detected in mutant roots at this time point, it can be suggested that the ongoing ethylene response is being counteracted by a higher conversion to ACCc, thereby confining the large peak in ACC levels observed after 24 h (Figure 5). Consistently, it was previously demonstrated that the conjugation of ACC into malonyl-ACC was stimulated by ethylene, which points to a feedback mechanism for ethylene biosynthesis [16].

It should be noted that, at the early time point (2 h), both WT and mutant plants accumulated similar Cd concentrations in their roots (Table 1). Therefore, the more severe oxidative challenge observed at the transcript level in roots of mutant plants can be attributed to the more pronounced constriction of the chelating and antioxidative capacity of GSH rather than to a difference in root Cd accumulation. At later time points (24 h and 72 h), GSH deficiency was associated with a lower Cd translocation to the aerial parts of the plants. Hendrix et al. (2020) indicated that limited GSH synthesis resulted in lowered PC synthesis and therefore impaired PC-dependent Cd translocation [50]. While our data further confirm these findings, it must be noted that early on during Cd exposure, Cd translocation was not restricted in the *cad2-1* mutant. To the contrary, Cd translocation was significantly higher compared to WT plants (Table 1). This possibly resulted from a transiently enhanced Cd chelation, as PC synthesis is activated by free Cd ions, which are, presumably, more abundant in the *cad2-1* mutant due to limiting GSH levels [51]. Concomitantly, we suggest that the maximum capacity of PC synthesis, and therefore Cd chelation, was reached more rapidly in mutant plants resulting in constricted Cd translocation at later time points (Table 1).

More pronounced Cd-induced stress responses could be expected from the fact that root GSH concentrations were still significantly lower in mutant plants compared to Cd-exposed WT plants after 24 h of exposure. However, for H_2_O_2_ levels this was not the case. Similar to the findings of Deckers et al. (2020), H_2_O_2_ levels peaked in roots of WT plants after 24 h [10]. In the mutant roots, however, this Cd-induced increase in H_2_O_2_ levels after 24 h was significantly smaller (Figure 3). This less pronounced Cd-induced response could be due to (1) the significantly higher basal H_2_O_2_ levels observed in mutant plants under control conditions, which were also mirrored by the higher expression of oxidative stress marker transcripts in non-exposed mutant plants (Table 3 and Figure S2); (2) a limited Cd-induced increase in ethylene production in the mutant plants after 24 h, as suggested above; and (3) activation of alternative antioxidative pathways like *APX* expression (Table S5). Based on these findings we suggest that, at the transcript level, the stress responses in the roots of *cad2-1* mutant plants after 24 h are largely similar to the WT root responses, but that the increased redox ratio and, therefore, stress levels are higher in mutant plants, which has repercussions later on. More specifically, after 72 h a significant decrease in root fresh weight was observed for the mutant genotype, while for WT roots this negative effect was limited and insignificant (Figure 1). At this later time point, oxidative stress was diminishing in WT plants, which could hint towards stabilisation, while the expression levels of oxidative stress markers as well as ethylene-related genes remained significantly more upregulated in mutant plants (Table 3 and Table 4).

In general, and in correspondence with earlier studies, responses are less severe and delayed in leaves of both WT and mutant plants compared to roots [5,10]. This observation is self-evident, as roots are the first organ to encounter Cd and are therefore exposed to higher levels of Cd in comparison to leaves. At the leaf level, the transcriptional responses were to a great extent similar between leaves of WT and mutant plants after 2 h of exposure, even though the leaf Cd concentration was significantly higher for mutant plants. Nevertheless, it seems that Cd levels were not sufficiently high to evoke clear differences in transcript responses. Noteworthy, however, is the induction of the redox-responsive transcription factor *RRTF1* (Table 3) that coincided with an increasing trend observed for H_2_O_2_ levels in mutant leaves and the significantly increased redox ratio observed after 2 h in mutant plants (Figure 3 and Figure 4). This points towards an increased early stress signalling in mutant leaves. However, as Cd accumulation was limited, this did not result in major responses. Nevertheless, after 24 h much clearer differences manifested at the transcripts level. More specifically, ethylene-related genes were induced to a significantly smaller extent in leaves of mutant plants in comparison to WT plants. This lower induction of, for example, ACC synthesis genes corresponded with the lower increase in leaf ACC concentrations observed in mutant plants after 24 h compared to WT plants (Table 4 and Figure 5a). While this observation can be explained by the limited translocation of Cd towards the aerial parts in mutant plants, especially after 24 h (Table 1), the limited production of ACC and possibly ethylene could impact signalling events that are needed for an optimal Cd-induced stress response. Based on these data and in correspondence with Schellingen et al. (2015), we can conclude that the WT ethylene response peaked after 24 h of Cd exposure and has a transient nature in leaves of WT plants [21]. Nevertheless, this peak in *ERF1* induction was not observed for mutant plants, not even after 72 h when the precursor levels increased to a similar extent as observed for the WT leaves after 24 h. This could possibly be attributable to limited Cd translocation and/or feedback on ethylene synthesis by conjugation of ACC. In line with the latter, ACCc levels also displayed a tendency to higher concentrations in the mutant plants (Figure 5b). Being a key regulator of the Cd-induced stress response, impaired timing and production of ethylene could disturb the stress response and therefore stabilisation followed by acclimation. While, after 72 h, H_2_O_2_ production was significantly higher in leaves of Cd-exposed WT plants, GSH concentrations were also enhanced in WT leaves, and consequently, no net increase in the H_2_O_2_/GSH ratio was observed (Figure 3 and Figure 4). This possibly points to a new equilibrium wherein enhanced GSH concentrations counteract the increase in H_2_O_2_, resulting in stabilisation and, later on, acclimation. Accordingly, Hendrix et al. (2020) demonstrated that leaf GSH concentrations remained elevated after 21 days of Cd exposure in WT plants. For mutant plants, however, the redox ratio was significantly elevated, as no stimulation in GSH concentrations was observed to compensate for the elevated H_2_O_2_ levels after 72 h. This lack in GSH stimulation was also observed in the *cad2-1* mutant after long-term exposure [50]. Tausz et al. (2004) stated that the inability to achieve acclimation, in this case by overcompensation of leaf GSH levels, will lead to degradation of the system [29]. Indeed, long-term exposure of the *cad2-1* mutant to Cd resulted in a significantly more pronounced chlorosis and negative impact on fresh weight and leaf surface area in comparison to WT plants [50]. In our study, it seemed that the short-term mutant leaf responses were similar to those of WT plants. This can be largely attributable to the limited Cd translocation in mutant plants at later time points (Table 1). However, the imbalance in redox state in the mutant, caused by the impaired GSH synthesising capacity, will eventually also lead to an exaggerated Cd effect at the level of the leaf [50].

## 5. Conclusions

Based on these data, we can conclude that during the early phase of the Cd-induced stress responses, the chelating capacity of GSH is prioritised over its antioxidative function at the root level, even in the GSH-deficient *cad2-1* mutant. Inevitably, this resulted in a more oxidised redox ratio (H_2_O_2_/GSH) and hence an increased oxidative challenge in the roots of mutant plants. Concomitantly, this early enhanced oxidative challenge triggered an accelerated response, which became apparent by the early induction of GSH synthesis genes and ACC production in mutant roots (Table 2 and Figure 5). While ACC concentrations were significantly higher in roots of Cd-exposed mutant plants compared to WT plants, this did not result in an enhanced ethylene response after 24 h. As mutant plants are naturally more challenged, and as a more rapid response could be observed after 2 h in the roots of mutant plants compared to WT plants, this suggests that signalling is accelerated in mutant roots and counteracted after 24 h by suppressing the stress hormone ethylene in order to prevent adverse effects and assure survival. Nevertheless, while the stress response at the root level peaked for WT plants after 24 h and declined afterwards, this was not the case for mutant plants. More specifically, oxidative-challenge-related genes and ethylene-responsive *ERF1* remained strongly upregulated up to 72 h after exposure in combination with significantly higher concentrations of ACC in mutant roots. This points to an ongoing stress response and lack of, or at least delayed, acclimation at the level of the roots. Responses in the leaves were largely defined by limited Cd translocation in the GSH-deficient mutant. As Cd levels are limited, the ethylene response was more confined, and while this response peaked after 24 h in WT plants, this was not the case for mutant leaves. We suggest that the limited induction of the ethylene response impairs proper stabilisation, for example, by the lack of stimulation in GSH metabolism at both the transcript and metabolite level. In addition, H_2_O_2_ levels increased to a similar extent in both genotypes, and although this was counterbalanced by the GSH stimulation observed in WT leaves, this was not the case for mutant plants, which resulted in a significantly elevated redox ratio after 72 h. Based on these findings, it can be concluded that, while responses were similar or even less pronounced in leaves of mutant plants compared to WT plants, the imbalance in redox status in *cad2-1* mutant plants and the lack of a proper transient ethylene signalling response will eventually lead to suboptimal acclimation and a more pronounced effect of Cd exposure. As ethylene-related responses were accelerated in the GSH-deficient mutant, these data further substantiate the interrelation between GSH and ethylene. To further unravel the reciprocity of both key regulators and, in general, the importance of ethylene in the Cd-induced stress response, it would be interesting to study these responses in ethylene signalling and biosynthesis mutant plants.

## Figures and Tables

**Table 1:** Root and leaf cadmium concentrations (mg kg^−1^ dry weight) and the corresponding Cd translocation factors (%) of 3-week-old wild-type (WT) and *cad2-1* mutant *Arabidopsis thaliana* plants grown under control conditions (0 µM CdSO_4_) or exposed to 5 µM CdSO_4_ during 2 h, 24 h, and 72 h. Data are presented as the mean ± S.E. of at least four biological independent replicates. Significant differences (*t*-test: *p* < 0.05) between both genotypes and within each time point are indicated with an asterisk (*). Data obtained from non-exposed (0 µM CdSO_4_) plants are not shown, as Cd concentrations were below the limit of detection.

**Cd Concentration (mg kg^−1^ DW)**							
		**2 h**		**24 h**		**72 h**	
	**[CdSO_4_]**	**WT**	***cad2-1***	**WT**	***cad2-1***	**WT**	***cad2-1***
*Leaf*	5 µM	1.75 ± 0.12	8.71 ± 1.09 *	1001.02 ± 15.36	275.20 ± 14.53 *	1492.14 ± 24.95	584.86 ± 17.23 *
*Root*	5 µM	1009.09 ± 28.66	1054.35 ± 20.62	2168.99 ± 52.39	3070.92 ± 52.39 *	2680.88 ± 92.24	3209.21 ± 88.32 *
**Translocation factor**							
		**2 h**		**24 h**		**72 h**	
	**[CdSO_4_]**	**WT**	***cad2-1***	**WT**	***cad2-1***	**WT**	***cad2-1***
	5 µM	0.17 ± 0.01	0.82 ± 0.01 *	46.21 ± 0.78	8.97 ± 0.49 *	55.89 ± 1.99	18.29 ± 0.81 *

**Table 2:** Relative gene expression levels of glutathione (GSH)-related genes in leaves and roots of 3-week-old wild-type (WT) and *cad2-1* mutant *Arabidopsis thaliana* plants grown under control conditions (0 µM CdSO_4_) or exposed to 5 µM CdSO_4_ during 2 h, 24 h, and 72 h. Data are presented as the mean ± S.E. of at least three biological independent replicates relative to the control of the corresponding genotype set at 1.00. Significant differences (2-way ANOVA: *p* < 0.05) between control and Cd-exposed plants of the same genotype within each time point are marked in colour (upregulated: aa, downregulated: aa). Significant differences between Cd-induced responses of both genotypes within each time point are indicated with an asterisk (*). *GSH1*: γ-glutamylcysteine synthetase, *GSH2*: GSH synthetase, *GR1*: glutathione reductase, *GGT1*: γ-glutamyl transpeptidase 1, *ZAT6*: zinc finger of *Arabidopsis thaliana* 6.

GSH-Related Genes							
		2 h		24 h		72 h	
Gene	CdSO_4_	WT	*cad2-1*	WT	*cad2-1*	WT	*cad2-1*
*Leaf*							
*GSH1*	0 µM	1.00 ± 0.03	1.00 ± 0.06	1.00 ± 0.14	1.00 ± 0.10	1.00 ± 0.12	1.00 ± 0.07
	5 µM	1.01 ± 0.07	0.92 ± 0.14	2.34 ± 0.12	2.30 ± 0.57	1.23 ± 0.09	0.84 ± 0.04 *
*GSH2*	0 µM	1.00 ± 0.06	1.00 ± 0.07	1.00 ± 0.14	1.00 ± 0.11	1.00 ± 0.10	1.00 ± 0.16
	5 µM	1.21 ± 0.03	1.06 ± 0.05	3.39 ± 0.31	3.14 ± 0.77	1.48 ± 0.05	2.07 ± 0.56
*GR1*	0 µM	1.00 ± 0.05	1.00 ± 0.07	1.00 ± 0.09	1.00 ± 0.12	1.00 ± 0.10	1.00 ± 0.09
	5 µM	0.89 ± 0.04	0.78 ± 0.02	3.00 ± 0.15	1.65 ± 0.29 *	1.23 ± 0.05	1.02 ± 0.05
*GGT1*	0 µM	1.00 ± 0.08	1.00 ± 0.06	1.00 ± 0.09	1.00 ± 0.19	1.00 ± 0.06	1.00 ± 0.17
	5 µM	0.86 ± 0.06	1.12 ± 0.07	2.98 ± 0.20	0.79 ± 0.12 *	1.76 ± 0.32	0.98 ± 0.10
*ZAT6*	0 µM	1.00 ± 0.16	1.00 ± 0.26	1.00 ± 0.27	1.00 ± 0.21	1.00 ± 0.04	1.00 ± 0.20
	5 µM	1.41 ± 0.34	2.10 ± 0.26	3.93 ± 0.71	1.85 ± 0.48	1.73 ± 0.66	1.13 ± 0.13
*Root*							
*GSH1*	0 µM	1.00 ± 0.05	1.00 ± 0.11	1.00 ± 0.02	1.00 ± 0.03	1.00 ± 0.09	1.00 ± 0.08
	5 µM	1.11 ± 0.03	1.25 ± 0.03	1.66 ± 0.12	2.15 ± 0.11 *	0.85 ± 0.05	0.96 ± 0.14
*GSH2*	0 µM	1.00 ± 0.09	1.00 ± 0.11	1.00 ± 0.04	1.00 ± 0.02	1.00 ± 0.08	1.00 ± 0.08
	5 µM	1.04 ± 0.03	1.39 ± 0.00	2.83 ± 0.08	2.67 ± 0.13	1.39 ± 0.23	1.78 ± 0.26
*GR1*	0 µM	1.00 ± 0.03	1.00 ± 0.14	1.00 ± 0.08	1.00 ± 0.03	1.00 ± 0.10	1.00 ± 0.04
	5 µM	0.98 ± 0.07	1.29 ± 0.02 *	2.17 ± 0.08	1.99 ± 0.05	1.74 ± 0.33	1.16 ± 0.12
*GGT1*	0 µM	1.00 ± 0.05	1.00 ± 0.01	1.00 ± 0.04	1.00 ± 0.05	1.00 ± 0.16	1.00 ± 0.10
	5 µM	1.43 ± 0.15	1.35 ± 0.03	2.85 ± 0.20	1.33 ± 0.05 *	1.08 ± 0.05	1.08 ± 0.09
*ZAT6*	0 µM	1.00 ± 0.13	1.00 ± 0.25	1.00 ± 0.17	1.00 ± 0.14	1.00 ± 0.14	1.00 ± 0.05
	5 µM	3.97 ± 0.48	23.05 ± 0.92 *	7.54 ± 0.81	10.89 ± 0.37	1.62 ± 0.22	9.48 ± 1.05 *

**Table 3:** Relative gene expression levels of oxidative-challenge-related genes in leaves and roots of 3-week-old wild-type (WT) and *cad2-1* mutant *Arabidopsis thaliana* plants grown under control conditions (0 µM CdSO_4_) or exposed to 5 µM CdSO_4_ during 2 h, 24 h, and 72 h. Data are presented as the mean ± S.E. of at least three biological independent replicates relative to the control of the corresponding genotype set at 1.00. Significant differences (2-way ANOVA: *p* < 0.05) between control and Cd-exposed plants of the same genotype, within each time point are marked in colour (upregulated: aa, downregulated: aa). Significant differences between Cd-induced responses of both genotypes within each time point are indicated with an asterisk (*). *ZAT12*: zinc finger of *Arabidopsis thaliana* 12, *RRTF1*: redox-responsive transcription factor 1.

Oxidative Challenge-Related Genes							
		2 h		24 h		72 h	
Gene	CdSO_4_	WT	*cad2-1*	WT	*cad2-1*	WT	*cad2-1*
*Leaf*							
*AT1G05340*	0 µM	1.00 ± 0.50	1.00 ± 0.29	1.00 ± 0.29	1.00 ± 0.34	1.00 ± 0.24	1.00 ± 0.12
	5 µM	0.41 ± 0.12	0.15 ± 0.04	32.29 ± 4.11	1.07 ± 0.42 *	1.17 ± 0.51	1.03 ± 0.21
*AT1G19020*	0 µM	1.00 ± 0.21	1.00 ± 0.27	1.00 ± 0.29	1.00 ± 0.34	1.00 ± 0.17	1.00 ± 0.11
	5 µM	0.92 ± 0.07	1.15 ± 0.08	9.45 ± 0.52	2.41 ± 0.59 *	2.00 ± 0.40	1.98 ± 0.48
*AT1G57630*	0 µM	1.00 ± 0.31	1.00 ± 0.30	1.00 ± 0.31	1.00 ± 0.38	1.00 ± 0.25	1.00 ± 0.16
	5 µM	0.57 ± 0.16	0.14 ± 0.05	9.88 ± 0.42	1.16 ± 0.22	2.26 ± 0.46	1.04 ± 0.23
*AT2G21640*	0 µM	1.00 ± 0.10	1.00 ± 0.08	1.00 ± 0.02	1.00 ± 0.32	1.00 ± 0.06	1.00 ± 0.20
	5 µM	1.11 ± 0.07	1.17 ± 0.05	12.72 ± 2.12	2.55 ± 0.38 *	2.68 ± 0.58	1.43 ± 0.08
*AT2G43510*	0 µM	1.00 ± 0.25	1.00 ± 0.18	1.00 ± 0.48	1.00 ± 0.18	1.00 ± 0.11	1.00 ± 0.19
	5 µM	1.21 ± 0.26	0.68 ± 0.03	20.04 ± 0.43	10.81 ± 3.70	3.19 ± 0.43	2.83 ± 0.40
*ZAT12*	0 µM	1.00 ± 0.22	1.00 ± 0.28	1.00 ± 0.35	1.00 ± 0.28	1.00 ± 0.25	1.00 ± 0.16
	5 µM	0.98 ± 0.21	1.86 ± 0.33	5.47 ± 0.58	2.07 ± 0.52 *	1.39 ± 0.37	1.31 ± 0.20
*RRTF1*	0 µM	1.00 ± 0.41	1.00 ± 0.61	1.00 ± 0.57	1.00 ± 0.35	1.00 ± 0.32	1.00 ± 0.24
	5 µM	0.62 ± 0.22	6.96 ± 1.57 *	0.39 ± 0.11	0.32 ± 0.12	1.71 ± 0.66	0.56 ± 0.27
*Root*							
*AT1G05340*	0 µM	1.00 ± 0.13	1.00 ± 0.14	1.00 ± 0.07	1.00 ± 0.06	1.00 ± 0.16	1.00 ± 0.12
	5 µM	1.35 ± 0.19	1.16 ± 0.10	1.54 ± 0.04	2.84 ± 0.19 *	1.09 ± 0.10	2.02 ± 0.22 *
*AT1G19020*	0 µM	1.00 ± 0.09	1.00 ± 0.18	1.00 ± 0.19	1.00 ± 0.29	1.00 ± 0.14	1.00 ± 0.21
	5 µM	6.66 ± 0.54	30.54 ± 2.30 *	21.94 ± 1.10	8.79 ± 0.68 *	4.34 ± 0.61	12.98 ± 1.05 *
*AT1G57630*	0 µM	1.00 ± 0.17	1.00 ± 0.02	1.00 ± 0.19	1.00 ± 0.19	1.00 ± 0.17	1.00 ± 0.17
	5 µM	1.00 ± 0.02	2.96 ± 0.21 *	39.49 ± 3.52	14.65 ± 0.97 *	6.80 ± 0.70	16.10 ± 1.14 *
*AT2G21640*	0 µM	1.00 ± 0.11	1.00 ± 0.14	1.00 ± 0.06	1.00 ± 0.08	1.00 ± 0.12	1.00 ± 0.13
	5 µM	1.13 ± 0.05	1.47 ± 0.04 *	6.49 ± 0.49	10.27 ± 1.10	1.40 ± 0.24	6.05 ± 0.47 *
*AT2G43510*	0 µM	1.00 ± 0.25	1.00 ± 0.25	1.00 ± 0.24	1.00 ± 0.12	1.00 ± 0.30	1.00 ± 0.21
	5 µM	2.48 ± 0.33	1.47 ± 0.08	25.89 ± 3.21	18.84 ± 1.68	158.69 ± 9.91	138.00 ± 16.19
*ZAT12*	0 µM	1.00 ± 0.21	1.00 ± 0.37	1.00 ± 0.24	1.00 ± 0.24	1.00 ± 0.15	1.00 ± 0.16
	5 µM	2.98 ± 0.29	12.88 ± 0.81 *	14.83 ± 0.04	10.04 ± 0.40	3.27 ± 0.30	36.59 ± 1.12 *
*RRTF1*	0 µM	1.00 ± 0.25	1.00 ± 0.60	1.00 ± 0.60	1.00 ± 0.42	1.00 ± 0.61	1.00 ± 0.49
	5 µM	1.22 ± 0.34	0.46 ± 0.10	6.67 ± 0.86	0.22 ± 0.02 *	1.18 ± 0.37	4.33 ± 0.31 *

**Table 4:** Relative gene expression levels of ethylene-related genes in leaves and roots of 3-week-old wild-type (WT) and *cad2-1* mutant *Arabidopsis thaliana* plants grown under control conditions (0 µM CdSO_4_) or exposed to 5 µM CdSO_4_ during 2 h, 24 h, and 72 h. Data are presented as the mean ± S.E. of at least three biological independent replicates relative to the control of the corresponding genotype set at 1.00. Significant differences (2-way ANOVA: *p* < 0.05) between control and Cd-exposed plants of the same genotype within each time point are marked in colour (upregulated: aa). Significant differences between Cd-induced responses of both genotypes within each time point are indicated with an asterisk (*). *ACS*: ACC synthase, *ACO*: ACC oxidase, *ERF1*: ethylene response factor 1, *OXI1*: oxidative signal inducible 1, *MPK*: mitogen-activated protein kinase, *WRKY33*: WRKY DNA-binding protein 33.

Ethylene-Related Genes							
		2 h		24 h		72 h	
Gene	CdSO_4_	WT	*cad2-1*	WT	*cad2-1*	WT	*cad2-1*
*Leaf*							
*ACS2*	0 µM	1.00 ± 0.38	1.00 ± 0.34	1.00 ± 0.32	1.00 ± 0.35	1.00 ± 0.23	1.00 ± 0.18
	5 µM	0.59 ± 0.21	0.34 ± 0.07	113.06 ± 13.61	16.22 ± 5.99 *	0.62 ± 0.21	1.52 ± 0.31
*ACS6*	0 µM	1.00 ± 0.34	1.00 ± 0.19	1.00 ± 0.14	1.00 ± 0.22	1.00 ± 0.16	1.00 ± 0.13
	5 µM	0.81 ± 0.44	2.70 ± 0.29 *	6.74 ± 1.07	1.17 ± 0.36 *	1.84 ± 0.27	2.80 ± 0.74 *
*ACO2*	0 µM	1.00 ± 0.03	1.00 ± 0.01	1.00 ± 0.08	1.00 ± 0.07	1.00 ± 0.14	1.00 ± 0.09
	5 µM	0.91 ± 0.05	0.96 ± 0.05	4.09 ± 0.77	6.25 ± 1.16	1.47 ± 0.14	1.39 ± 0.02
*ACO4*	0 µM	1.00 ± 0.13	1.00 ± 0.15	1.00 ± 0.08	1.00 ± 0.61	1.00 ± 0.09	1.00 ± 0.11
	5 µM	0.96 ± 0.03	0.80 ± 0.05	6.49 ± 0.49	2.18 ± 0.44 *	2.37 ± 0.15	1.66 ± 0.19
*ERF1*	0 µM	1.00 ± 0.19	1.00 ± 0.23	1.00 ± 0.15	1.00 ± 0.29	1.00 ± 0.24	1.00 ± 0.13
	5 µM	1.57 ± 0.15	1.23 ± 0.21	38.31 ± 6.76	9.18 ± 2.49 *	2.99 ± 0.55	4.53 ± 0.77
*OXI1*	0 µM	1.00 ± 0.23	1.00 ± 0.20	1.00 ± 0.35	1.00 ± 0.22	1.00 ± 0.17	1.00 ± 0.08
	5 µM	1.22 ± 0.14	1.10 ± 0.26	32.46 ± 4.95	3.92 ± 0.89 *	1.70 ± 0.54	1.87 ± 0.09
*MPK3*	0 µM	1.00 ± 0.12	1.00 ± 0.15	1.00 ± 0.18	1.00 ± 0.24	1.00 ± 0.13	1.00 ± 0.10
	5 µM	1.08 ± 0.01	0.99 ± 0.09	1.80 ± 0.07	0.54 ± 0.04 *	2.01 ± 0.33	1.20 ± 0.16
*MPK6*	0 µM	1.00 ± 0.07	1.00 ± 0.11	1.00 ± 0.11	1.00 ± 0.90	1.00 ± 0.03	1.00 ± 0.15
	5 µM	0.88 ± 0.05	0.86 ± 0.03	2.26 ± 0.23	0.98 ± 0.12 *	1.21 ± 0.15	0.96 ± 0.11
*WRKY33*	0 µM	1.00 ± 0.34	1.00 ± 0.24	1.00 ± 0.30	1.00 ± 0.26	1.00 ± 0.11	1.00 ± 0.25
	5 µM	1.41 ± 0.34	1.25 ± 0.41	3.69 ± 0.39	0.93 ± 0.25 *	1.19 ± 0.28	1.42 ± 0.25
*Root*							
*ACS2*	0 µM	1.00 ± 0.10	1.00 ± 0.12	1.00 ± 0.18	1.00 ± 0.24	1.00 ± 0.09	1.00 ± 0.13
	5 µM	1.29 ± 0.10	1.25 ± 0.08	14.22 ± 1.63	108.08 ± 0.74 *	6.29 ± 0.1	23.74 ± 1.68 *
*ACS6*	0 µM	1.00 ± 0.18	1.00 ± 0.23	1.00 ± 0.18	1.00 ± 0.24	1.00 ± 0.14	1.00 ± 0.08
	5 µM	3.22 ± 0.77	10.08 ± 1.14 *	14.22 ± 1.63	13.02 ± 0.74	3.98 ± 0.52	12.22 ± 1.58 *
*ACO2*	0 µM	1.00 ± 0.12	1.00 ± 0.09	1.00 ± 0.07	1.00 ± 0.03	1.00 ± 0.10	1.00 ± 0.05
	5 µM	0.88 ± 0.09	1.28 ± 0.12	2.07 ± 0.21	1.98 ± 0.06	1.08 ± 0.10	0.96 ± 0.13
*ACO4*	0 µM	1.00 ± 0.10	1.00 ± 0.15	1.00 ± 0.05	1.00 ± 0.05	1.00 ± 0.00	1.00 ± 0.06
	5 µM	2.61 ± 0.14	3.71 ± 0.26	6.99 ± 0.39	6.26 ± 0.22	3.07 ± 0.29	4.13 ± 0.33
*ERF1*	0 µM	1.00 ± 0.13	1.00 ± 0.18	1.00 ± 0.15	1.00 ± 0.07	1.00 ± 0.12	1.00 ± 0.09
	5 µM	3.37 ± 0.27	39.93 ± 2.37 *	137.31 ± 19.52	69.26 ± 5.58 *	13.55 ± 2.44	79.92 ± 7.98 *
*OXI1*	0 µM	1.00 ± 0.10	1.00 ± 0.19	1.00 ± 0.06	1.00 ± 0.22	1.00 ± 0.14	1.00 ± 0.13
	5 µM	3.44 ± 0.35	16.77 ± 1.46 *	7.75 ± 1.12	12.54 ± 0.10	1.81 ± 0.19	11.55 ± 0.84 *
*MPK3*	0 µM	1.00 ± 0.03	1.00 ± 0.03	1.00 ± 0.05	1.00 ± 0.12	1.00 ± 0.16	1.00 ± 0.02
	5 µM	2.28 ± 0.09	6.41 ± 0.36 *	3.50 ± 0.9	5.82 ± 0.27 *	2.00 ± 0.24	3.06 ± 0.23
*MPK6*	0 µM	1.00 ± 0.03	1.00 ± 0.13	1.00 ± 0.05	1.00 ± 0.01	1.00 ± 0.12	1.00 ± 0.12
	5 µM	1.81 ± 0.38	2.46 ± 0.02	2.23 ± 0.03	1.49 ± 0.27 *	0.79 ± 0.05	0.82 ± 0.07
*WRKY33*	0 µM	1.00 ± 0.11	1.00 ± 0.17	1.00 ± 0.09	1.00 ± 0.22	1.00 ± 0.23	1.00 ± 0.17
	5 µM	5.01 ± 0.84	19.71 ± 1.02 *	8.47 ± 0.59	21.07 ± 1.24 *	2.77 ± 0.32	7.16 ± 0.76 *

## Data Availability

All of the data is contained within the article and the Supplementary Materials.

## Supplementary Materials

The following are available online at https://www.mdpi.com/article/10.3390/antiox11010006/s1, Figure S1: Relative hydrogen peroxide (H_2_O_2_) levels (control WT set at 1.00), Figure S2: Relative gene expression levels of a subset of oxidative stress markers, Table S1: Forward and reverse primer sequences used for gene expression analyses of genes of interest, Table S2: Forward and reverse primer sequences used for gene expression analyses of reference genes, Table S3: Reverse transcription quantitative PCR (RT-qPCR) parameters according to the Minimum Information for publication of Quantitative real-time PCR Experiments, Table S4: Percentage reduced glutathione (GSH) deduced from oxidised GSSG levels and total GSH levels, Table S5: Relative gene expression levels of antioxidative enzymes, Table S6: Relative gene expression levels of ROS-generating NADPH oxidases.

## References

[1] Jarup L., Akesson A. (2009). Current status of cadmium as an environmental health problem. Toxicol. Appl. Pharm..

[2] Khan M.A., Khan S., Khan A., Alam M. (2017). Soil contamination with cadmium, consequences and remediation using organic amendments. Sci. Total Environ..

[3] Verbruggen N., Hermans C., Schat H. (2009). Mechanisms to cope with arsenic or cadmium excess in plants. Curr. Opin. Plant Biol..

[4] Clemens S., Aarts M.G., Thomine S., Verbruggen N. (2013). Plant science: The key to preventing slow cadmium poisoning. Trends Plant Sci..

[5] Jozefczak M., Keunen E., Schat H., Bliek M., Hernandez L.E., Carleer R., Remans T., Bohler S., Vangronsveld J., Cuypers A. (2014). Differential response of Arabidopsis leaves and roots to cadmium: Glutathione-related chelating capacity vs antioxidant capacity. Plant Physiol. Biochem..

[6] Cuypers A., Plusquin M., Remans T., Jozefczak M., Keunen E., Gielen H., Opdenakker K., Nair A.R., Munters E., Artois T.J. (2010). Cadmium stress: An oxidative challenge. Biometals.

[7] Gill S.S., Anjum N.A., Hasanuzzaman M., Gill R., Trivedi D.K., Ahmad I., Pereira E., Tuteja N. (2013). Glutathione and glutathione reductase: A boon in disguise for plant abiotic stress defense operations. Plant Physiol. Biochem..

[8] Jozefczak M., Remans T., Vangronsveld J., Cuypers A. (2012). Glutathione is a key player in metal-induced oxidative stress defenses. Int. J. Mol. Sci..

[9] Zagorchev L., Seal C.E., Kranner I., Odjakova M. (2013). A central role for thiols in plant tolerance to abiotic stress. Int. J. Mol. Sci..

[10] Deckers J., Hendrix S., Prinsen E., Vangronsveld J., Cuypers A. (2020). Identifying the Pressure Points of Acute Cadmium Stress Prior to Acclimation in Arabidopsis thaliana. Int. J. Mol. Sci..

[11] Cobbett C.S., May M.J., Howden R., Rolls B. (1998). The glutathione-deficient, cadmium-sensitive mutant, cad2-1, of Arabidopsis thaliana is deficient in gamma-glutamylcysteine synthetase. Plant J..

[12] Hothorn M., Wachter A., Gromes R., Stuwe T., Rausch T., Scheffzek K. (2006). Structural basis for the redox control of plant glutamate cysteine ligase. J Biol. Chem..

[13] Keunen E., Schellingen K., Vangronsveld J., Cuypers A. (2016). Ethylene and metal stress: Small molecule, big impact. Front. Plant Sci..

[14] Argueso C.T., Hansen M., Kieber J.J. (2007). Regulation of ethylene biosynthesis. J. Plant Growth Regul..

[15] Vanderstraeten L., Van Der Straeten D. (2017). Accumulation and Transport of 1-Aminocyclopropane-1-Carboxylic Acid (ACC) in Plants: Current Status, Considerations for Future Research and Agronomic Applications. Front. Plant Sci..

[16] Van de Poel B., Van Der Straeten D. (2014). 1-aminocyclopropane-1-carboxylic acid (ACC) in plants: More than just the precursor of ethylene!. Front. Plant Sci..

[17] Schellingen K., Van Der Straeten D., Vandenbussche F., Prinsen E., Remans T., Vangronsveld J., Cuypers A. (2014). Cadmium-induced ethylene production and responses in Arabidopsis thaliana rely on ACS2 and ACS6 gene expression. BMC Plant Biol..

[18] Opdenakker K., Remans T., Keunen E., Vangronsveld J., Cuypers A. (2012). Exposure of Arabidopsis thaliana to Cd or Cu excess leads to oxidative stress mediated alterations in MAPKinase transcript levels. Environ. Exp. Bot..

[19] Rentel M.C., Lecourieux D., Ouaked F., Usher S.L., Petersen L., Okamoto H., Knight H., Peck S.C., Grierson C.S., Hirt H. (2004). OXI1 kinase is necessary for oxidative burst-mediated signalling in *Arabidopsis*. Nature.

[20] Remans T., Opdenakker K., Smeets K., Mathijsen D., Vangronsveld J., Cuypers A. (2010). Metal-specific and NADPH oxidase dependent changes in lipoxygenase and NADPH oxidase gene expression in *Arabidopsis thaliana* exposed to cadmium or excess copper. Funct. Plant Biol..

[21] Schellingen K., Van Der Straeten D., Remans T., Vangronsveld J., Keunen E., Cuypers A. (2015). Ethylene signalling is mediating the early cadmium-induced oxidative challenge in *Arabidopsis thaliana*. Plant Sci.

[22] Yoshida S., Tamaoki M., Ioki M., Ogawa D., Sato Y., Aono M., Kubo A., Saji S., Saji H., Satoh S. (2009). Ethylene and salicylic acid control glutathione biosynthesis in ozone-exposed *Arabidopsis thaliana*. Physiol. Plant..

[23] Chen H.J., Huang C.S., Huang G.J., Chow T.J., Lin Y.H. (2013). NADPH oxidase inhibitor diphenyleneiodonium and reduced glutathione mitigate ethephon-mediated leaf senescence, H_2_O_2_ elevation and senescence-associated gene expression in sweet potato (*Ipomoea batatas*). J. Plant Physiol..

[24] Zhang Y., He Q., Zhao S., Huang L., Hao L. (2014). *Arabidopsis* ein2-1 and npr1-1 response to Al stress. Bull. Environ. Contam. Toxicol..

[25] Smeets K., Ruytinx J., Van Belleghem F., Semane B., Lin D., Vangronsveld J., Cuypers A. (2008). Critical evaluation and statistical validation of a hydroponic culture system for *Arabidopsis thaliana*. Plant Physiol. Biochem..

[26] Keunen E., Truyens S., Bruckers L., Remans T., Vangronsveld J., Cuypers A. (2011). Survival of Cd-exposed Arabidopsis thaliana: Are these plants reproductively challenged?. Plant Physiol. Biochem..

[27] Remans T., Keunen E., Bex G.J., Smeets K., Vangronsveld J., Cuypers A. (2014). Reliable Gene Expression Analysis by Reverse Transcription-Quantitative PCR: Reporting and Minimizing the Uncertainty in Data Accuracy. Plant Cell.

[28] Bustin S.A., Benes V., Garson J.A., Hellemans J., Huggett J., Kubista M., Mueller R., Nolan T., Pfaffl M.W., Shipley G.L. (2009). The MIQE guidelines: Minimum information for publication of quantitative real-time PCR experiments. Clin. Chem..

[29] Tausz M., Šircelj H., Grill D. (2004). The glutathione system as a stress marker in plant ecophysiology: Is a stress-response concept valid?. J. Exp. Bot..

[30] Krznaric E., Verbruggen N., Wevers J.H.L., Carleer R., Vangronsveld J., Colpaert J.V. (2009). Cd-tolerant *Suillus luteus*: A fungal insurance for pines exposed to Cd. Environ. Pollut..

[31] Jozefczak M., Bohler S., Schat H., Horemans N., Guisez Y., Remans T., Vangronsveld J., Cuypers A. (2015). Both the concentration and redox state of glutathione and ascorbate influence the sensitivity of Arabidopsis to cadmium. Annals of Botany.

[32] Jozefczak M. (2014). Deficiency in Ascorbate is Compensated by Glutathione in Cadmium-Exposed Arabidopsis Mutants but Glutathione Deficiency Demands for Multiple Alternatives. Ph.D. Thesis.

[33] Cuypers A., Hendrix S., dos Reis R.A., De Smet S., Deckers J., Gielen H., Jozefczak M., Loix C., Vercampt H., Vangronsveld J. (2016). Hydrogen peroxide, signaling in disguise during metal phytotoxicity. Front. Plant Sci..

[34] Gadjev I., Vanderauwera S., Gechev T.S., Laloi C., Minkov I.N., Shulaev V., Apel K., Inze D., Mittler R., Van Breusegem F. (2006). Transcriptomic footprints disclose specificity of reactive oxygen species signaling in *Arabidopsis*. Plant Physiol..

[35] Dubreuil-Maurizi C., Vitecek J., Marty L., Branciard L., Frettinger P., Wendehenne D., Meyer A.J., Mauch F., Poinssot B. (2011). Glutathione Deficiency of the *Arabidopsis* Mutant pad2-1 Affects Oxidative Stress-Related Events, Defense Gene Expression, and the Hypersensitive Response. Plant Physiol..

[36] Han Y., Chaouch S., Mhamdi A., Queval G., Zechmann B., Noctor G. (2013). Functional analysis of *Arabidopsis* mutants points to novel roles for glutathione in coupling H_2_O_2_ to activation of salicylic acid accumulation and signaling. Antioxid. Redox Signal..

[37] Mhamdi A., Hager J., Chaouch S., Queval G., Han Y., Taconnat L., Saindrenan P., Gouia H., Issakidis-Bourguet E., Renou J.P. (2010). Arabidopsis GLUTATHIONE REDUCTASE1 plays a crucial role in leaf responses to intracellular hydrogen peroxide and in ensuring appropriate gene expression through both salicylic acid and jasmonic acid signaling pathways. Plant Physiol..

[38] Chen J., Yang L., Yan X., Liu Y., Wang R., Fan T., Ren Y., Tang X., Xiao F., Liu Y. (2016). Zinc-Finger Transcription Factor ZAT6 Positively Regulates Cadmium Tolerance through the Glutathione-Dependent Pathway in *Arabidopsis*. Plant Physiol..

[39] Liu X.M., Nguyen X.C., Kim K.E., Han H.J., Yoo J., Lee K., Kim M.C., Yun D.J., Chung W.S. (2013). Phosphorylation of the zinc finger transcriptional regulator ZAT6 by MPK6 regulates Arabidopsis seed germination under salt and osmotic stress. Biochem. Biophys. Res. Commun..

[40] Rizhsky L., Davletova S., Liang H.J., Mittler R. (2004). The zinc finger protein Zat12 is required for cytosolic ascorbate peroxidase 1 expression during oxidative stress in *Arabidopsis*. J. Biol. Chem..

[41] Han G.L., Lu C.X., Guo J.R., Qiao Z.Q., Sui N., Qiu N.W., Wang B.S. (2020). C2H2 Zinc Finger Proteins: Master Regulators of Abiotic Stress Responses in Plants. Front. Plant Sci..

[42] Schellingen K., Van Der Straeten D., Remans T., Loix C., Vangronsveld J., Cuypers A. (2015). Ethylene biosynthesis is involved in the early oxidative challenge induced by moderate Cd exposure in *Arabidopsis thaliana*. Environ. Exp. Bot..

[43] Datta R., Kumar D., Sultana A., Hazra S., Bhattacharyya D., Chattopadhyay S. (2015). Glutathione Regulates 1-Aminocyclopropane-1-Carboxylate Synthase Transcription via WRKY33 and 1-Aminocyclopropane-1-Carboxylate Oxidase by Modulating Messenger RNA Stability to Induce Ethylene Synthesis during Stress. Plant Physiol..

[44] Montero-Palmero M.B., Martín-Barranco A., Escobar C., Hernández L.E. (2014). Early transcriptional responses to mercury: A role for ethylene in mercury-induced stress. New Phytol..

[45] Skottke K.R., Yoon G.M., Kieber J.J., DeLong A. (2011). Protein Phosphatase 2A Controls Ethylene Biosynthesis by Differentially Regulating the Turnover of ACC Synthase Isoforms. PLoS Genet..

[46] Li G., Meng X., Wang R., Mao G., Han L., Liu Y., Zhang S. (2012). Dual-Level Regulation of ACC Synthase Activity by MPK3/MPK6 Cascade and Its Downstream WRKY Transcription Factor during Ethylene Induction in *Arabidopsis*. PLoS Genet..

[47] Yoo S.D., Cho Y.H., Sheen J. (2009). Emerging connections in the ethylene signaling network. Trends Plant Sci..

[48] Liu Y., Zhang S. (2004). Phosphorylation of 1-Aminocyclopropane-1-Carboxylic Acid Synthase by MPK6, a Stress-Responsive Mitogen-Activated Protein Kinase, Induces Ethylene Biosynthesis in *Arabidopsis*. Plant Cell.

[49] Mersmann S., Bourdais G., Rietz S., Robatzek S. (2010). Ethylene Signaling Regulates Accumulation of the FLS2 Receptor and Is Required for the Oxidative Burst Contributing to Plant Immunity. Plant Physiol..

[50] Hendrix S., Jozefczak M., Wojcik M., Deckers J., Vangronsveld J., Cuypers A. (2020). Glutathione: A key player in metal chelation, nutrient homeostasis, cell cycle regulation and the DNA damage response in cadmium-exposed *Arabidopsis thaliana*. Plant Physiol. Biochem..

[51] Ogawa S., Yoshidomi T., Yoshimura E. (2011). Cadmium(II)-stimulated enzyme activation of *Arabidopsis thaliana* phytochelatin synthase 1. J. Inorg. Biochem..

