# Supplementary material for: Glutathione Is Required for the Early Alert Response and Subsequent Acclimation in Cadmium-Exposed Arabidopsis thaliana Plants
Source: Antioxidants (Basel). 2021 Dec 21;11(1):6. doi: 10.3390/antiox11010006 (PMC8773091; doi:10.3390/antiox11010006)
Supplement: Supplementary file 1 [file antioxidants-11-00006-s001.zip › antioxidants-1494438-supplementary.pdf]

Supplemental Table S1 Forward and reverse primer sequences used for gene expression analyses (RT-qPCR) of genes of interest. Ex jn: exon junction x2: primer concentration of 600 nM instead of 300 nM

Genes of interest					
**Gene**	**Locus**	**Forward Primer (5′- 3′)**	**Reverse Primer (5′-3′)**	**Primer Location**	**Amplicon Size**
GSH1	AT4G23100	CCCTGGTGAACTGCCTTCA	CATCAGCACCTCTCATCTCCA	Exon 10 and 11	101 bp
GSH2	AT5G27380	GGACTCGTCGTTGGTGACAA	TCTGGGAATGCAGTTGGTAGC	Exon 1 and 2	101 bp
GR1	AT3G24170	CTCAAGTGTGGAGCAACCAAAG	ATGCGTCTGGTCACACTGC	Exon 15 and 16	101 bp
GGT1	AT4G39640	TAGATGTTCCACCACCAGCA	GGCAATAATGTTGGCTCCTC	Exon 4 and 5	140 bp
ZAT6	AT5G04340	TGACCTGCCTTCTTCTTCGT	GTCCACCAAGAGCCTGGTAA	No introns	113 bp
RBOHC	AT5G51060	TCACCAGAGACTGGCACAATAAA	GATGCTCGACCTGAATGCTC	Exon 6 and 7	101 bp
RBOHD	AT5G47910	AACTCTCCGCTGATTCCAACG	TGGTCAGCGAAGTCTTTAGATTCCT	Exon 1 and 2	91 bp
RBOHF	AT1G64060	GGTGTCATGAACGAAGTTGCA	AATGAGAGCAGAACGAGCATCA	Exon 11 and 12	105 bp
AT1G05340	AT1G05340	TCGGTAGCTCAGGGTAAAGTGG	CCAGGGCACAACAGCAACA	Exon 2 and 3	91 bp
AT1G19020	AT1G19020	GAAAATGGGACAAGGGTTAGACAAA	CCCAACGAAAACCAATAGCAGA	No introns	92 bp
AT1G57630	AT1G57630	ACTCAAACAGGCGATCAAAGGA	CACCAATTCGTCAAGACAACACC	No introns	91 bp
AT2G21640	AT2G21640	GACTTGTTTCAAAAACACCATGGAC	CACTTCCTTAGCCTCAATTTGCTTC	Exon 1 and 2	91 bp
AT2G43510	AT2G43510	ATGGCAAAGGCTATCGTTTCC	CGTTACCTTGCGCTTCTATCTCC	Exon 1 and 2	91 bp
ZAT12	AT5G59820	GTGCGAGTCACAAGAAGCCTAACA	GCGACGACGTTTTCACCTTCTTCA	No introns	72 bp
RRTF1	AT4G34410	CGGAGCAAGAGCTTTCAGTT	GCGCTTATCACTGTGCTGTC	No introns	109 bp
ACS2	AT1G01480	CATGTTCTGCCTTGCGGATC	ACCTGTCCGCCACCTCAAGT	Exon 3 and 4	91 bp

ACS6 [x2]	AT4G11280	TTAGCTAATCCCGGCGATGG	ACAAGATTCACTCCGGTTCTCCA	Exon 3 and 4	92 bp
ACO2	AT1G62380	TCTACGTTCGTCACCTCCCTCA	CTCTTACCAAAGTCTTTCATGGCC	Exon 2 and 3	91 bp
ACO4	AT1G05010	CTCCGATGTCCCTGATCTCG	ATCCAGTAGCTCCTCCGACAACT	Exon 2 and 3	91 bp
ERF1 [x2]	AT3G23240	TCCTCGGCGATTCTCAATTTT	CAACCGGAGAACAACCATCCT	No introns	91 bp
OXI1	AT3G25250	TAGAGGATCGAACCGGAAAG	GACCCTTGATTTCCTCAACG	Exon 2	149 bp
MPK3	AT3G45640	GACGTTTGACCCCAACAGAA	TGGCTTTTGACAGATTGGCTC	Exon 5 and 6	103 bp
MPK6	AT2G43790	TAAGTTCCCGACAGTGCATCC	GATGGGCCAATGCGTCTAA	Exon 5 and 6	101 bp
WRKY33	AT2G38470	TCATCGATTGTCAGCAGAGACG	CCATTCCCACCATTTGTTTCAT	Exon 3 and 4	92 bp
APX1	AT1G07890	TGCCACAAGGATAGGTCTGG	CCTTCCTTCTCTCCGCTCAA	Exon 5 and 6	101 bp
APX2	AT3G09640	TTGCTGTTGAGATCACTGGAGGA	TGAGGCAGACGACCTTCAGG	Exon 3 and 4	91 bp
CAT1	AT1G20630	AAGTGCTTCATCGGGAAGGA	CTTCAACAAAACGCTTCACGA	Ex jn 5, 6 and exon 7	103 bp
CAT2	AT4G35090	AACTCCTCCATGACCGTTGGA	TCCGTTCCCTGTCGAAATTG	Exon 2 and 3	76 bp

Supplmental Table S2 Forward and reverse primer sequences used for gene expression analyses (RT-qPCR) of selected reference genes. Ex jn: exon junction; UTR: untranslated region

		Reference genes			
**Gene**	**Locus**	**Forward Primer (5′- 3′)**	**Reverse Primer (5′-3′)**	**Primer Location**	**Amplicon Size**
Leaf **2 h**					
PPR	AT5G55840	AAGACAGTGAAGGTGCAACCTTACT	AGTTTTTGAGTTGTATTTGTCAGAGAAAG	UTR	59 bp
MON1	AT5G55840	AACTCTATGCAGCATTTGATCCACT	TGATTGCATATCTTTATCGCCATC	Exon 13 and 14	61 bp

EF1α	AT5G60390	TGAGCACGCTCTTCTTGCTTTCA	GGTGGTGGCATCCATCTTGTTACA	Exon 1 and ex jn 1,2	76 bp
**24 h**					
UBC21	AT5G25760	CTGCGACTCAGGGAATCTTCTAA	TTGTGCCATTGAATTGAACCC	Ex jn 3,4 and exon 4	61 bp
PPR	AT5G55840	AAGACAGTGAAGGTGCAACCTTACT	AGTTTTTGAGTTGTATTTGTCAGAGAAAG	UTR	59 bp
MON1	AT5G55840	AACTCTATGCAGCATTTGATCCACT	TGATTGCATATCTTTATCGCCATC	Exon 13 and 14	61 bp
**72 h**					
ACT2	AT3G18780	CTTGCACCAAGCAGCATGAA	CCGATCCAGACACTGTACTTCCTT	Exon 2	68 bp
FBOX	AT5G15710	TTTCGGCTGAGAGGTTCGAGT	GATTCCAAGACGTAAAGCAGATCAA	Exon 1	63 bp
UBQ10	AT4G05320	GGCCTTGTATAATCCCTGATGAATAAG	AAAGAGATAACAGGAACGGAAACATAGT	Exon 1 and ex jn 1,2	76 bp
Root					
**2 h**					
MON1	AT5G55840	AACTCTATGCAGCATTTGATCCACT	TGATTGCATATCTTTATCGCCATC	Exon 13 and 14	61 bp
RHIP1	AT4G26410	GAGCTGAAGTGGCTTCCATGAC	GGTCCGACATACCCATGATCC	Ex jn 7, 8 and exon 8	81 bp
EF1α	AT5G60390	TGAGCACGCTCTTCTTGCTTTCA	GGTGGTGGCATCCATCTTGTTACA	Exon 1 and 1,2	76 bp
**24 h**					
UBC21	AT5G25760	CTGCGACTCAGGGAATCTTCTAA	TTGTGCCATTGAATTGAACCC	Exon 3,4 and 4	61 bp
MON1	AT5G55840	AACTCTATGCAGCATTTGATCCACT	TGATTGCATATCTTTATCGCCATC	Exon 13 and 14	61 bp
TIP41-like	AT4G34270	GTGAAAACTGTTGGAGAGAAGCAA	TCAACTGGATACCCTTTCGCA	Exon 7 and Exon 7,8	61 bp
YSL8	AT5G08290	TTACTGTTTCGGTTGTTCTCCATTT	CACTGAATCATGTTCGAAGCAAGT	UTR	61 bp
UBQ10	AT4G05320	GGCCTTGTATAATCCCTGATGAATAAG	AAAGAGATAACAGGAACGGAAACATAGT	Exon 1 and ex jn 1,2	76 bp
EF1α	AT5G60390	TGAGCACGCTCTTCTTGCTTTCA	GGTGGTGGCATCCATCTTGTTACA	Exon 1 and ex jn 1,2	76 bp
**72 h**					
UBC21	AT5G25760	CTGCGACTCAGGGAATCTTCTAA	TTGTGCCATTGAATTGAACCC	Exon 3,4 and 4	61 bp

UBQ10	AT4G05320	GGCCTTGTATAATCCCTGATGAATAAG	AAAGAGATAACAGGAACGGAAACATAGT	Exon 1 and 1,2	76 bp
EF1α	AT5G60390	TGAGCACGCTCTTCTTGCTTTCA	GGTGGTGGCATCCATCTTGTTACA	Exon 1 and 1,2	76 bp

Table S3 Reverse transcription quantitative PCR (RT-qPCR) parameters according to the Minimum Information for publication of Quantitative real-time PCR Experiments (MIQE) guidelines based on Bustin *et al.* (2009).

**Sample - Template**	
SOURCE	roots and leaves of three-weeks-old *Arabidopsis thaliana* plants grown in hydroponics
METHOD OF PRESERVATION	snap frozen in N_2, long-term storage at -80°
STORAGE TIME	< 6 months
HANDLING	frozen
EXTRACTION METHOD	silica-columns: RNAqueous™ Kit (Ambion, Thermo Fisher Scientific, Waltham, MA, USA)
RNA; DNA-FREE	turbo DNA-*free*™ Kit (Ambion, Thermo Fisher Scientific, Waltham, MA, USA)
	use of intron-spanning primers if possible
	verification of amplicon-specificity via dissociation curve
CONCENTRATION	NanoDrop® ND-1000 spectrophotometer (Thermo Fisher Scientific, Waltham, Ma, USA)
**Assay optimisation – Validation**	
ACCESSION NUMBER	see Supplementary Table S1 & S2
AMPLICON DETAILS	see Supplementary Table S1 & S2
PRIMERS SEQUENCE	see Supplementary Table S1 & S2
IN SILICO	TAIR primer-BLAST http://www.arabidopsis.org/Blast/index.jsp
EMPIRICAL	primer concentration of 300 nM unless mentioned otherwise (Table S1 & S2)
	annealing temperature of 60°C
PRIMING CONDITIONS	combinations of oligo-dT and random hexamer primers
PCR EFFICIENCY	dilution curves of pooled samples (slope, deviation)
LINEAR DYNAMIC RANGE	samples are within the range of the efficiency curve
**RT and qPCR**	
PROTOCOLS	Turbo DNA-*free*™ Kit (Ambion, Thermo Fisher Scientific, Waltham, MA, USA)
	PrimeScript™ RT Reagent Kit (Perfect Real Time, Takara Bio Inc., Kusatsu, Japan)
	Qauntinova™ SYBR® Green PCR kit (Qiagen, Hilde, Germany)

	as described in the Materials and Methods section
REAGENTS	as described in the Materials and Methods section
NON TEMPLATE CONTROL	verification based on Cq-value and dissociation curve
**Data analysis**	
SOFTWARE	QuantStudio™ Design & Analysis Software (Applied Biosystems, Thermo Fisher Scientific, Foster City, CA, USA)
STATISTICAL ANALYSIS	specified in Materials and Methods section and legends of Figures and Tables
NORMALISATION	a selection of at least three reference genes based on the GrayNorm algoritm (Remans *et al.* 2014)
	see Materials and Methods section

Supplemental Table S4 Percentage reduced glutathione (GSH) deduced from oxidised GSSG levels and total GSH levels in leaves and roots of 3 weeks old wild-type (WT) and *cad2-1* mutant *Arabidopsis thaliana* plants grown under control conditions (0 μM $CdSO_4$, light bars) or exposed to 5 μM $CdSO_4$ (dark bars) during 2 h, 24 h and 72 h. For each time point, data presented as the mean ± S.E. of 4 biological replicates. Significant differences (2-way ANOVA: $p < 0.05$) within each time point are indicated with different letters. L.O.D.: limit of detection for oxidised GSSG.

		**Reduced GSH (%)**					
		**2 h**		**24 h**		**72 h**	
	**[$CdSO_4$]**	**WT**	***cad2-1***	**WT**	***cad2-1***	**WT**	***cad2-1***
Leaf	0 μM	96.87 ± 9.01^{a}	97.98 ± 1.74^{a}	96.94 ± 0.30^{a}	96.11 ± 0.75^{a}	90.82 ± 0.81^{a}	94.37 ± 0.46^{b}
	5 μM	97.56 ± 7.43^{a}	98.06 ± 1.23^{a}	98.92 ± 0.22^{b}	98.84 ± 0.07^{ab}	96.56 ± 1.08^{c}	98.08 ± 0.43^{c}
Root	0 μM	95.95 ± 7.38	96.92 ± 1.91	97.97 ± 0.39^{a}	98.05 ± 0.24^{a}	97.00 ± 0.92^{a}	96.89 ± 1.33^{a}
	5 μM	99.96 ± 0.22	L.O.D.	97.34 ± 0.70^{ab}	95.52 ± 0.09^{b}	98.06 ± 0.11^{a}	96.77 ± 1.03^{a}

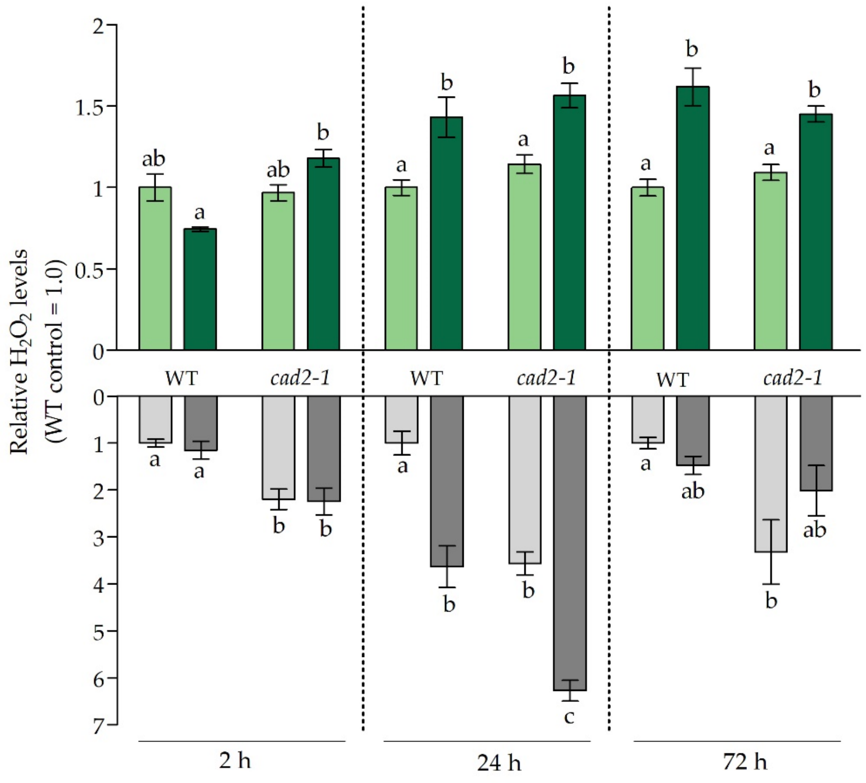

Supplemental Figure S1 Relative hydrogen peroxide (H_2O_2) levels in leaves (green bars) and roots (grey bars) of 3 weeks old wild-type (WT) and *cad2-1* mutant *Arabidopsis thaliana* plants grown under control conditions (0 µM $CdSO_4$, light bars) or exposed to 5 µM $CdSO_4$ (dark bars) during 2 h, 24 h and 72 h. Data are presented as the mean ± S.E. of at least three biological independent replicates relative to the control of the WT set at 1.00. Significant differences (2-way ANOVA: $p < 0.05$) within each time point are indicated with different letters.

Supplemental Table S5 Relative gene expression levels of antioxidative enzymes in leaves and roots of 3 weeks old wild-type (WT) and *cad2-1* mutant *Arabidopsis thaliana* plants grown under control conditions (0 µM $CdSO_4$, light bars) or exposed to 5 µM $CdSO_4$ (dark bars) during 2 h, 24 h and 72 h. Data are presented as the mean ± S.E. of at least three biological independent replicates relative to the control of the corresponding genotype set at 1.00. Significant differences (2-way ANOVA: $p < 0.05$) between control and Cd-exposed plants of the same genotype, within each time point are marked in colour (upregulated: [green], downregulated: [red]). Significant differences between Cd-induced responses of both genotypes whithin each time point are indicated with an asterisk (*). *APX*: ascorbate peroxidase , *CAT:* catalase.

		Antioxidative enzymes					
		2 h		24 h		72 h	
**Gene**	**$CdSO_4$**	**WT**	***cad2-1***	**WT**	***cad2-1***	**WT**	***cad2-1***
Leaf							
APX1	0 µM	1.00 ± 0.08	1.00 ± 0.08	1.00 ± 0.02	1.00 ± 0.08	1.00 ± 0.02	1.00 ± 0.02
	5 µM	1.04 ± 0.12	0.65 ± 0.04*	2.13 ± 0.18	1.25 ± 0.27	1.41 ± 0.36	0.44 ± 0.00
APX2	0 µM	1.00 ± 0.09	1.00 ± 0.15	1.00 ± 0.21	1.00 ± 0.35	1.00 ± 0.12	1.00 ± 0.29
	5 µM	0.65 ± 0.09	0.85 ± 0.18	4.21 ± 1.41	0.41 ± 0.20*	4.02 ± 0.75	0.22 ± 0.00
CAT1	0 µM	1.00 ± 0.07	1.00 ± 0.14	1.00 ± 0.04	1.00 ± 0.14	1.00 ± 0.18	1.00 ± 0.22
	5 µM	1.10 ± 0.14	0.62 ± 0.05*	3.48 ± 0.41	1.20 ± 0.36*	1.12 ± 0.34	0.91 ± 0.03
CAT2	0 µM	1.00 ± 0.03	1.00 ± 0.12	1.00 ± 0.10	1.00 ± 0.10	1.00 ± 0.01	1.00 ± 0.06
	5 µM	0.89 ± 0.11	0.71 ± 0.05	0.30 ± 0.04	0.72 ± 0.03*	1.02 ± 0.22	0.66 ± 0.10
Root							
APX1	0 µM	1.00 ± 0.01	1.00 ± 0.11	1.00 ± 0.05	1.00 ± 0.03	1.00 ± 0.14	1.00 ± 0.09
	5 µM	1.10 ± 0.04	1.16 ± 0.05	1.04 ± 0.05	1.75 ± 0.21	0.98 ± 0.05	0.75 ± 0.05
APX2	0 µM	1.00 ± 0.19	1.00 ± 0.04	1.00 ± 0.02	1.00 ± 0.02	1.00 ± 0.17	1.00 ± 0.30
	5 µM	2.25 ± 0.27	14.95 ± 1.61*	9.02 ± 1.29	157.47 ± 36.74*	0.26 ± 0.07	6.99 ± 0.62*
CAT1	0 µM	1.00 ± 0.05	1.00 ± 0.02	1.00 ± 0.07	1.00 ± 0.04	1.00 ± 0.14	1.00 ± 0.06
	5 µM	0.90 ± 0.08	0.89 ± 0.04	3.74 ± 0.25	4.16 ± 0.07	1.81 ± 0.02	2.64 ± 0.33*
CAT2	0 µM	1.00 ± 0.15	1.00 ± 0.08	1.00 ± 0.04	1.00 ± 0.08	1.00 ± 0.15	1.00 ± 0.07
	5 µM	0.78 ± 0.12	0.78 ± 0.00	0.38 ± 0.01	1.05 ± 0.04*	0.64 ± 0.02	0.36 ± 0.05*

Supplemental Table S6 Relative gene expression levels of ROS-generating NADPH oxidases in leaves and roots of 3 weeks old wild-type (WT) and *cad2-1* mutant *Arabidopsis thaliana* plants grown under control conditions (0 µM $CdSO_4$, light bars) or exposed to 5 µM $CdSO_4$ (dark bars) during 2 h, 24 h and 72 h. Data are presented as the mean ± S.E. of at least three biological independent replicates relative to the control of the corresponding genotype set at 1.00. Significant differences (2-way ANOVA: $p < 0.05$) between control and Cd-exposed plants of the same genotype, within each time point are marked in colour (upregulated: green, downregulated: red). Significant differences between Cd-induced responses of both genotypes whithin each time point are indicated with an asterisk (*). *RBOH*: respiratory burst oxidase homologue.

		ROS-generating NADPH oxidase					
		2 h		24 h		72 h	
**Gene**	**$CdSO_4$**	**WT**	***cad2-1***	**WT**	***cad2-1***	**WT**	***cad2-1***
Leaf							
RBOHC	0 µM	1.00 ± 0.32	1.00 ± 0.33	1.00 ± 0.14	1.00 ± 0.46	1.00 ± 0.15	1.00 ± 0.38
	5 µM	0.47 ± 0.06	0.74 ± 0.34	79.81 ± 6.35	0.63 ± 0.11*	0.58 ± 0.14	1.42 ± 0.42
RBOHD	0 µM	1.00 ± 0.02	1.00 ± 0.04	1.00 ± 0.16	1.00 ± 0.23	1.00 ± 0.05	1.00 ± 0.06
	5 µM	1.37 ± 0.02	1.17 ± 0.04*	2.45 ± 0.09	1.46 ± 0.23	2.25 ± 0.28	1.93 ± 0.19
RBOHF	0 µM	1.00 ± 0.30	1.00 ± 0.19	1.00 ± 0.08	1.00 ± 0.13	1.00 ± 0.10	1.00 ± 0.14
	5 µM	0.72 ± 0.13	0.58 ± 0.07	6.74 ± 0.82	0.66 ± 0.11*	3.99 ± 0.58	1.20 ± 0.01*
Root							
RBOHC	0 µM	1.00 ± 0.01	1.00 ± 0.17	1.00 ± 0.06	1.00 ± 0.01	1.00 ± 0.16	1.00 ± 0.10
	5 µM	1.41 ± 0.19	1.18 ± 0.03	1.04 ± 0.06	0.87 ± 0.06	0.68 ± 0.02	0.69 ± 0.04
RBOHD	0 µM	1.00 ± 0.10	1.00 ± 0.14	1.00 ± 0.04	1.00 ± 0.05	1.00 ± 0.11	1.00 ± 0.14
	5 µM	1.56 ± 0.01	2.43 ± 0.13*	4.50 ± 0.60	3.90 ± 0.21	2.51 ± 0.28	2.38 ± 0.45
RBOHF	0 µM	1.00 ± 0.03	1.00 ± 0.03	1.00 ± 0.03	1.00 ± 0.04	1.00 ± 0.08	1.00 ± 0.06
	5 µM	1.28 ± 0.03	1.28 ± 0.05	2.20 ± 0.08	1.92 ± 0.05	1.98 ± 0.25	1.78 ± 0.23

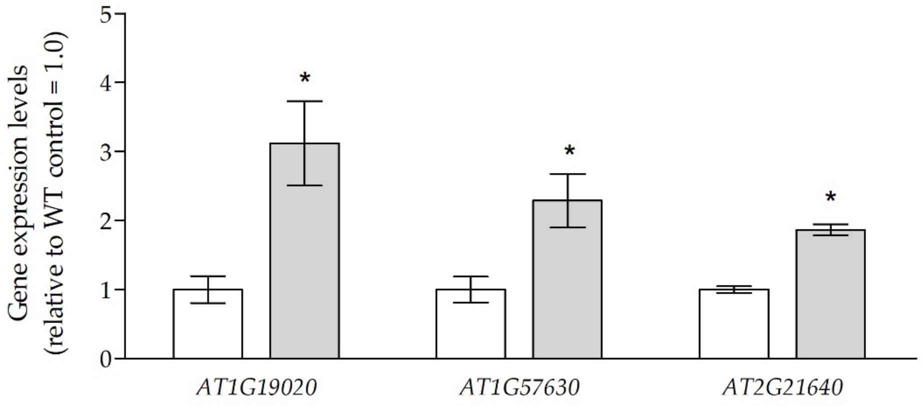

Supplemental Figure S2 Relative gene expression levels of a subset of oxidative stress markers in roots after 24 h under control conditions (0 µM $CdSO_4$) in 3 weeks old wild-type (WT, white bars) and *cad2-1* mutant (grey bars) *Arabidopsis thaliana* plants. Data are represented as the mean ± S.E. of at least three biological independent replicates relative to the control of the WT set at 1.00. For each gene, significant differences (t-test: $p < 0.05$) between the two genotypes under control conditions are indicated with an asterisk (*).
